# Bounds on the Sum-Rate of MIMO Causal Source Coding Systems with Memory under Spatio-Temporal Distortion Constraints

**DOI:** 10.3390/e22080842

**Published:** 2020-07-30

**Authors:** Photios A. Stavrou, Jan Østergaard, Mikael Skoglund

**Affiliations:** 1 Department of Intelligent Systems, Division of Information Science and Engineering, KTH Royal Institute of Technology, 11428 Stockholm, Sweden; skoglund@kth.se; 2 Section on Signal and Information Processing, Department of Electronic Systems, Aalborg University, 9000 Aalborg, Denmark; jo@es.aau.dk

**Keywords:** bounds, causal coding, one-shot information theory, convex programming, estimation, spatial distortion constraints, temporal distortion constraints, multi-user rate distortion theory

## Abstract

In this paper, we derive lower and upper bounds on the OPTA of a two-user multi-input multi-output (MIMO) causal encoding and causal decoding problem. Each user’s source model is described by a multidimensional Markov source driven by additive i.i.d. noise process subject to three classes of spatio-temporal distortion constraints. To characterize the lower bounds, we use state augmentation techniques and a data processing theorem, which recovers a variant of rate distortion function as an information measure known in the literature as nonanticipatory ϵ-entropy, sequential or nonanticipative RDF. We derive lower bound characterizations for a system driven by an i.i.d. Gaussian noise process, which we solve using the SDP algorithm for all three classes of distortion constraints. We obtain closed form solutions when the system’s noise is possibly non-Gaussian for both users and when only one of the users is described by a source model driven by a Gaussian noise process. To obtain the upper bounds, we use the best linear forward test channel realization that corresponds to the optimal test channel realization when the system is driven by a Gaussian noise process and apply a sequential causal DPCM-based scheme with a feedback loop followed by a scaled ECDQ scheme that leads to upper bounds with certain performance guarantees. Then, we use the linear forward test channel as a benchmark to obtain upper bounds on the OPTA, when the system is driven by an additive i.i.d. non-Gaussian noise process. We support our framework with various simulation studies.

## 1. Problem Statement

**Figure 1:**
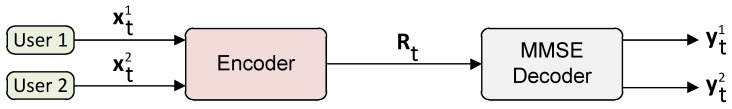
System model. The encoder receives information from two users that do not interact from a dynamical system perspective, but they are allowed to allocate bits between them and across the dimensions. The compression is done causally whereas the clocks of the encoder and the decoder are assumed to be synchronized.

We consider the two-user causal encoding and causal decoding setup illustrated in Figure 1. In this setup, users 1 and 2 are modeled by the following discrete-time time-invariant multidimensional Markov processes:

(1) $$\begin{array}{c} \begin{array}{cc} x_{t+1}^{1} & =A_{1}x_{t}^{1}+w_{t}^{1},\;t=0,1,\ldots \\ x_{t+1}^{2} & =A_{2}x_{t}^{2}+w_{t}^{2}, \end{array}, \end{array}$$

where $x_{t}^{1}\in R^{p_{1}},x_{t}^{2}\in R^{p_{2}}$, with $p_{1}$ not necessarily equal to $p_{2}$, $\left(A_{1},A_{2}\right)$ are known constant matrices of appropriate dimensions and $\left(w_{t}^{1},w_{t}^{2}\right)$ are additive i.i.d. possibly non-Gaussian noise processes with zero mean and covariance matrix $\Sigma_{w^{i}}≻0,i=1,2$, independent of $x_{0}^{i},\;i=1,2$ and from each other for all t≥0. The initial states $x_{0}^{i},\;i=1,2$ are given by $x_{0}^{i}∼\left(0;\Sigma_{x_{0}^{i}}\right),\;i=1,2$. Finally, we restrict the eigenvalues of $\left(A_{1},A_{2}\right)$ to be within the unit circle, which means that each user’s system model in (1) is asymptotically stable (i.e., asymptotically stationary).

The goal is to cast the performance of the setup in Figure 1 under various distortion metrics when the encoder compress information causally whereas the lossless compression between the encoder and the decoder is done in one shot assuming their clocks are synchronized.

First, we apply state space augmentation [1] to the state-space models in (1) to transform them into a single augmented state-space model as follows:

(2) $$\begin{array}{c} x_{t+1}=Ax_{t}+w_{t}, \end{array}$$

where $x_{t+1}={\left[x_{t+1}^{1^{T}},x_{t+1}^{2^{T}}\right]}^{{}^{T}}\in R^{p_{1}+p_{2}}$, *A* is a block diagonal matrix and $w_{t}$ is an additive i.i.d. possibly non-Gaussian noise process such that $w_{t}∼\left(0;\Sigma_{w}\right)$ where $\left(A,\Sigma_{w}\right)$ are of the form

(3) $$\begin{array}{c} \left[\begin{array}{cc} A_{1} & 0\\ 0 & A_{2} \end{array}\right]\in R^{\left(p_{1}+p_{2}\right)\times \left(p_{1}+p_{2}\right)},\;\left[\begin{array}{cc} \Sigma_{w^{1}} & 0\\ 0 & \Sigma_{w^{2}} \end{array}\right]\in R^{\left(p1+p2)\times (p1+p2\right)}. \end{array}$$

We note that the operation in (3) can be mathematically denoted as $A=A_{1}⊕A_{2}$ and similarly, $\Sigma_{w}=\Sigma_{w^{1}}⊕\Sigma_{w^{2}}$ (see the notation section for “⊕”).

**System Operation**: The encoder at each time instant *t* observes the augmented state $x_{t}$ and generates the data packet $\ell_{t}\in \left\{1,\ldots ,2^{R_{t}}\right\}$ of instantaneous rate $R_{t}$. At time *t*, the packet $\ell_{t}$ is sent over a *noiseless channel* with rate $R_{t}$. The decoder at each time *t*, receives $\ell_{t}$ to construct an estimate $y_{t}$ of $x_{t}$. We assume that the clocks of the encoder and decoder are synchronized. Formally, the encoder (E) and the decoder (D) are specified by a sequence of measurable functions $\left\{\left(f_{t},g_{t}\right):\;t\in N_{0}\right\}$ as follows:

(4) $$\begin{array}{c} \begin{array}{cc} E:\;\ell_{t} & =f_{t}\left(\ell^{t-1},x^{t}\right),\;\ell_{-\infty }^{-1}=\emptyset ,\\ D:\;y_{t} & =g_{t}\left(\ell^{t}\right). \end{array} \end{array}$$

### 1.1. Generalizations

It should be noted that the setup in Figure 1 can be generalized to any finite number of users. The only change will appear in the number of state-space equations and the dimension of the vectors and matrices in the augmented state-space representation of (2).

Next, we explain the setup of two users that are correlated (in states). In such scenario, users 1 and 2 are modeled by the following discrete-time time-invariant multidimensional Markov processes:

(5) $$\begin{array}{c} \begin{array}{cc} x_{t+1}^{1} & =A_{11}x_{t}^{1}+A_{12}x_{t}^{2}+w_{t}^{1},\;t=0,1,\ldots \\ x_{t+1}^{2} & =A_{22}x_{t}^{2}+A_{21}x_{t}^{1}+w_{t}^{2}, \end{array}, \end{array}$$

where $x_{t}^{1}\in R^{p_{1}},x_{t}^{2}\in R^{p_{2}}$, with $p_{1}$ not necessarily equal to $p_{2}$, $\left(A_{11},\;A_{12},\;A_{21},\;A_{22}\right)$ are known constant matrices of appropriate dimensions whereas all the other assumptions remain similar to the user models described in (1). The single augmented state-space model now is obtained as follows:

(6) $$\begin{array}{c} x_{t+1}=\hat{A}x_{t}+w_{t}, \end{array}$$

where $\hat{A}$ is a block matrix of the form

(7) $$\begin{array}{c} \left[\begin{array}{cc} A_{11} & A_{12}\\ A_{21} & A_{22} \end{array}\right]\in R^{\left(p_{1}+p_{2}\right)\times \left(p_{1}+p_{2}\right)}, \end{array}$$

where $A_{11},\;A_{22}$ are square matrices but $\left(A_{12},\;A_{21}\right)$ may be rectangular matrices (if $p_{1}\neq p_{2}$). We will not consider this case in our paper because it is straightforward by replacing everywhere matrix *A* with matrix $\hat{A}$. Clearly, this case can be generalized to any finite number of users with appropriate modifications on the state-space models.

### 1.2. Distortion Constraints

In this work we consider three types of distortion constraints. These are articulated as follows:

(i) a per-dimension (spatial) distortion constraint on the asymptotically averaged total (across the time) MMSE covariance matrix;
(ii) an asymptotically averaged total (across the time and across the space) distortion constraint;
(iii) a covariance matrix distortion constraint.

Next, we give the definition of each distortion constraint and explain some of their utility in multi-user systems.

A per-dimension (spatial) distortion constraint imposed on the covariance distortion matrix $\Sigma_{\Delta }≜{lim sup}_{n⟶\infty }\frac{1}{n+1}\sum_{t=0}^{n}E\left\{\left(x_{t}-y_{t}\right){\left(x_{t}-y_{t}\right)}^{T}\right\}$, where $\Sigma_{\Delta }⪰0$, is defined as follows:

(8) $$\begin{array}{c} \Sigma_{\Delta_{ii}}\leq D_{ii},\;i=1,\ldots ,p, \end{array}$$

where $D_{ii}\in \left[0,D_{ii}^{\max}\right]$ are given diagonal entries of the positive semidefinite matrix $\hat{D}⪰0$, with $\mathrm{trace}(\hat{D})\equiv D$, $D\in [0,D_{\max}]$. Note that under this distortion constraint, it trivially holds that ${lim sup}_{n⟶\infty }\frac{1}{n+1}\sum_{t=0}^{n}E\left\{\left|\right|x_{t}-y_{t}{\left|\right|}_{2}^{2}\right\}\leq D$.

**Figure 2:**
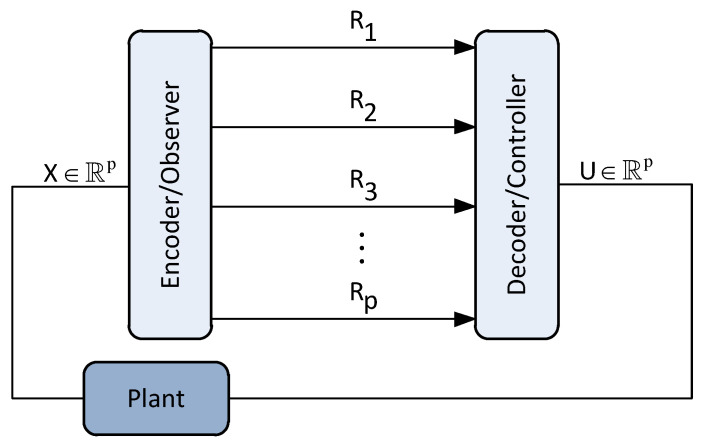
Centralized multivariable multi-input multi-output (MIMO) control system.

**Utility:** The choice of per-dimension distortion constraints is arguably more realistic in various network systems. For instance, one use of such hard constraints can be found in multivariable feedback control systems also called centralized multi-input multi-output (MIMO) systems [2] (see Figure 2). In such networks, it may be the case that one wishes to minimize the temporally total performance criterion or satisfy some total fidelity constraint. However, in addition it is always required that the resource allocation to the different nodes (or variables) to never exceed certain performance thresholds when the demands in data transmission within the communication link allows only limited rate. Nonetheless, the problem is that variables interact. Some variables could be considered more important for certain applications according to the demands of the system or the quality of service, which is why they need hard constraints.

An asymptotically averaged total (across the time and space) distortion constraint is defined as follows:

(9) $$\begin{array}{c} \underset{n⟶\infty }{lim sup}\frac{1}{n+1}\sum_{t=0}^{n}E\left\{\left|\right|x_{t}-y_{t}{\left|\right|}_{2}^{2}\right\}\leq D_{{}^{T}}, \end{array}$$

where $D_{{}^{T}}\in \left[0,D_{{}^{T}}^{\max}\right]$, with $D_{{}^{T}}$ not necessarily equal to *D*.

**Utility:** The asymptotically averaged total (across the time and space) distortion constraint ensure shared or allocated distortion arbitrarily among the transmit dimensions. The combination of the per-dimension distortion constraint with the averaged total distortion constraints ensure a total allocated distortion budget in the system that depends on the allowable (by design) distortion budget at each dimension (or user).

A covariance matrix distortion constraint is a generalization of the per-dimension distortion constraint defined by

(10) $$\begin{array}{c} \Sigma_{\Delta }⪯D_{\mathrm{cov}}, \end{array}$$

where $D_{\mathrm{cov}}⪰0$.

**Utility:** During the recent years, there has been a shift from conventional MSE distortion constraints (scalar-valued target distortions) to covariance matrix distortions in the areas of multiterminal and distributed source coding [3,4,5,6,7] and signal processing [7,8,9]. Nonetheless, the argument for considering covariance distortion constraints despite its difficulty is its generality and the flexibility in formulating new problems. For instance, one practical example would be wireless adhoc microphones, that transmit to receiver(s) over an MIMO channel. In such setups, perhaps the receiver(s) need to do beam forming or some multi-channel Wiener filtering variants. In both cases, one needs to know the covariance matrix of e.g., the error signal (covariance distortion matrix) to perform the desired signal enhancement. For example, if the quality of one of the signals is too bad, this could harm the overall signal enhancement, and one therefore need to trade-off the bits correctly among the microphones. In an adhoc microphone array, the different signals are naturally correlated, which adds an interesting interplay between them that goes beyond MSE distortion.

### 1.3. Operational Interpretations

In this subsection, we use the three types of distortion constraints introduced in Section 1.2 to define the corresponding operational definitions for which we study lower and upper bounds in this paper.

**Definition** **1**  (Causal RDF subject to (8)). *The operational causal RDF under per-dimension distortion constraints is defined as follows:*

(11) $$\begin{array}{c} R_{\mathrm{pd}}^{c}\left(D\right)≜\underset{\begin{array}{c} \left(f_{t},g_{t}\right):\;t\in N_{0}\\ \Sigma_{\Delta_{ii}}\leq D_{ii},\;i=1,\ldots ,p \end{array}}{\inf}\underset{n⟶\infty }{lim sup}\frac{1}{n+1}\sum_{t=0}^{n}R_{t}, \end{array}$$

*where $D_{ii}\in \left[0,D_{ii,\max}\right]$ and $D\in [0,D_{\max}]$.*

**Definition** **2**  (Causal RDF subject to (8) and (9)). *The operational causal RDF under joint per-dimension and asymptotically averaged total distortion constraints is defined as follows:*

(12) $$\begin{array}{c} R_{\mathrm{joint}}^{c}\left(D^{*}\right)≜\underset{\begin{array}{c} \left(f_{t},g_{t}\right):\;t\in N_{0}\\ \Sigma_{\Delta_{ii}}\leq D_{ii},\;i=1,\ldots ,p,\\ {lim sup}_{n⟶\infty }\frac{1}{n+1}\sum_{t=0}^{n}E\left\{\left|\right|x_{t}-y_{t}{\left|\right|}_{2}^{2}\right\}\leq D^{*} \end{array}}{\inf}\underset{n⟶\infty }{lim sup}\frac{1}{n+1}\sum_{t=0}^{n}R_{t}, \end{array}$$

where $D^{*}=\min\left\{D_{{}^{T}},D\right\}$.

**Interplay between Definitions 1 and 2.** Clearly, Definition 1 is a lower bound to Definition 2 because its constraint set of feasible solutions is larger. Note that, in general, the asymptotically averaged total distortion constraint in (12) is active when $D_{{}^{T}}\leq D$, otherwise, it is a trivial constraint and (12) is equivalent to the optimization problem of (11). This observation will be shown via a simulation study in the sequel of the paper.

**Definition** **3**  (Causal RDF subject to (10)). *The operational causal RDF under covariance matrix distortion constraints is defined as follows:*

(13) $$\begin{array}{c} R^{c}\left(D_{\mathrm{cov}}\right)≜\underset{\begin{array}{c} \left(f_{t},g_{t}\right):\;t\in N_{0}\\ \Sigma_{\Delta }⪯D_{\mathrm{cov}} \end{array}}{\inf}\underset{n⟶\infty }{lim sup}\frac{1}{n+1}\sum_{t=0}^{n}R_{t}, \end{array}$$

*where $D_{\mathrm{cov}}⪰0$.*

**Literature Review.** In information theory, causal coding and causal decoding also termed zero-delay coding (see, e.g., [10,11,12,13,14]) does not rely on the traditional construction based on random codebooks that in turn allows asymptotically large source vector dimensions [15] to establish achievability of a certain (non-causal) rate-distortion performance. Indeed, the optimal rate-distortion performance for causal source coding (with the clocks of the encoder and decoder to be synchronized), is hard to compute and often bounds are derived in the literature. For example, lower and upper bounds on the operational causal RDF subject to solely the distortion constraint in (9) (or the more stringent per instant distortion constraint $E\{||x_{t}-y_{t}{\left|\right|}_{2}^{2}\}\geq D_{t},\;\forall t$) are already studied extensively for various special cases of the setup of Figure 1, see, e.g., [11,14,16,17] and the references therein. In this work, we study new problems related to the causal RDF for the general multi-user source coding setup of Figure 1 under various classes of distortion constraints that their utility (partly explained in Section 1.2) has not been studied in the literature so far. These bounds are established using tools from information theory, convex optimization and causal MMSE estimation.

### 1.4. Contributions

In this paper we obtain the following results for the setup of Figure 1.

- Characterization and computation of the true lower bounds on (11)–(13) when the users’ augmented source model is driven by a Gaussian noise process (Lemma 3, Theorem 2).
- Analytical lower bounds on (11)–(13) when the users’ augmented source model is driven by additive i.i.d. noise process (including both additive Gaussian and non-Gaussian noise) (Theorem 3). As a consequence, we also obtain analytical lower bounds when only one of the users’ source model is driven by a Gaussian noise process (Corollary 2).
- Characterizations and computation of achievable bounds on (11)–(13) when the users’ augmented source model is driven by a Gaussian noise process (Theorem 4).
- Characterizations of achievable bounds on (11)–(13), when the users’ augmented source model is driven by additive i.i.d. non-Gaussian noise process (Theorem 5).

Machinery and tools. The information theoretic rate distortion definitions that are used to obtain the lower bounds in this paper are derived using a data processing theorem (Theorem 1) that reveals the “suitable” information measure to use. The derivation of the steady-state characterization of the lower bounds in Lemma 3 is derived using inequalities from matrix algebra and a convexity argument that allows the use of Jensen’s inequality. To derive lower bounds beyond additive i.i.d. Gaussian noise process, we use the fact that the characterizations of the lower bounds for the Gaussian case are in fact the characterizations obtained for the best linear coding policies (Lemma 4) hence these can serve as a benchmark to derive lower bounds beyond Gaussian noise process by leveraging certain trace/determinant inequalities and most importantly Minkowski’s determinant inequality ([18], Exercise 12.13) and EPI [19]. The upper bounds on the OPTA when the system’s noise is Gaussian, are derived using a causal sequential DPCM-based scheme with feedback loop which is equivalent to the scheme first derived in [14], followed by an ECDQ scheme that uses vector quantization. The upper bounds on the OPTA, when the system’s noise is additive i.i.d. non-Gaussian are obtained using precisely the same trick that is used to obtain the lower bounds, i.e., we use the linear test channel realization that achieves similar upper bounds for the Gaussian case and then, using an SLB type concept (Theorem 5) we obtain the desired results.

The paper is structured as follows. In Section 2 we characterize and compute lower bounds on the OPTA of (11)–(13). In Section 3 we characterize and compute achievable coding scheme on the OPTA of (11)–(13). We draw conclusions and future directions in Section 4.

## 2. Lower Bounds

In this section, we first choose a suitable information measure that will be used to derive a lower bound on Definitions 1–3. This information measure is a variant of directed information subject to some conditional independence constraints. Then, we obtain lower bounds on Definitions 1–3 for jointly Gaussian Markov processes and for Markov processes driven by additive i.i.d. possibly non-Gaussian noise process.

First, we write the joint distribution of the communication system of Figure 1, i.e., from the two users described by the augmented state $\left\{x_{t}:\;t\in N_{0}^{n}\right\}$ to the augmented output of the MMSE decoder $\left\{y_{t}:t\in N_{0}^{n}\right\}$. In particular, the joint distribution induced by the joint process $\left\{\left(x_{t},\ell_{t},y_{t}\right):\;t\in N_{0}^{n}\right\}$ admits the following decomposition:

(14) $$\begin{array}{cc} P(dx^{n},d\ell^{n},dy^{n}) & ={⊗}_{t=0}^{n}P\left(dy_{t},d\ell_{t},dx_{t}|y^{t-1},\ell^{t-1},x^{t-1}\right)\\ \; & ={⊗}_{t=0}^{n}P\left(dy_{t}|y^{t-1},x^{t},\ell^{t}\right)⊗P\left(d\ell_{t}|\ell^{t-1},y^{t-1},x^{t}\right)⊗P\left(dx_{t}|x^{t-1},y^{t-1},\ell^{t-1}\right)-a.s.\\ \; & ={⊗}_{t=0}^{n}P\left(dy_{t}|y^{t-1},\ell^{t}\right)⊗P\left(d\ell_{t}|\ell^{t-1},y^{t-1},x^{t}\right)⊗P\left(dx_{t}|x^{t-1}\right)-a.s., \end{array}$$

which means that the augmented state “source” process $x_{t}$, and the decoder’s output process $y_{t}$ satisfy the following conditional independence constraints:

(15) $$\begin{array}{cc} P(dx_{t}|x^{t-1},y^{t-1},\ell^{t-1}) & =P(dx_{t}|x^{t-1})-a.s., \end{array}$$

(16) $$\begin{array}{cc} P(dy_{t}|y^{t-1},\ell^{t},x^{t}) & =P(dy_{t}|y^{t-1},\ell^{t})-a.s. \end{array}$$

For (14) we state the following technical remark.

**Remark** **1**  (Trivial initial information). *In (14) we assume that the joint distribution $P(dx^{-1},d\ell^{-1},dy^{-1})$ generates trivial information. This means that $P\left(dx_{0}|x^{-1},y^{-1},\ell^{-1}\right)=P\left(dx_{0}\right)$, $P\left(dy_{0}|y^{-1},x^{0},\ell^{0}\right)=P\left(dy_{0}|x_{0},\ell_{0}\right)$ and $P\left(d\ell_{0}|\ell^{-1},y^{-1},x^{0}\right)=P\left(d\ell_{0}|x_{0}\right)$.*

We next prove a data processing theorem.

**Theorem** **1**  (Data processing theorem). *Provided the decomposition of the joint distribution in (14) holds, the augmented state-space representation of the system in Figure 1 admits the following data processing inequalities:*

(17) $$\begin{array}{c} \begin{array}{c} I\left(x^{n};y^{n}\right)\overset{\left(\mathrm{ii}\right)}{\leq }I(x^{n};\ell^{n}\left|\right|y^{n-1})\overset{\left(i\right)}{\leq }\sum_{t=0}^{n}R_{t}, \end{array} \end{array}$$

*where*

(18) $$\begin{array}{c} \begin{array}{cc} I(x^{n};\ell^{n}||y^{n-1}) & ≜\sum_{t=0}^{n}I\left(x^{t};\ell_{t}|\ell^{t-1},y^{t-1}\right),\\ I(x^{n};y^{n}) & ≜\sum_{t=0}^{n}I\left(x^{t};y_{t}|y^{t-1}\right), \end{array} \end{array}$$

*assuming $I(x^{t};\ell_{t}|\ell^{t-1},y^{t-1})<\infty ,\;\forall t,$ and $I(x^{t};y_{t}|y^{t-1})<\infty ,\;\forall t$.*

**Proof.**  We first prove **(i)**.

$$\begin{array}{cc} \sum_{t=0}^{n}R_{t} & \geq \sum_{t=0}^{n}H\left(\ell_{t}|\ell^{t-1}\right)\\ \; & \overset{\left(a\right)}{\geq }\sum_{t=0}^{n}H\left(\ell_{t}|\ell^{t-1},y^{t-1}\right)\\ \; & \overset{\left(b\right)}{\geq }\sum_{t=0}^{n}\left[H\left(\ell_{t}|\ell^{t-1},y^{t-1}\right)-H\left(\ell_{t}|\ell^{t-1},y^{t-1},x^{t}\right)\right]\\ \; & \overset{\left(c\right)}{=}\sum_{t=0}^{n}I\left(x^{t};\ell_{t}|\ell^{t-1},y^{t-1}\right)\\ \; & \equiv I(x^{n};\ell^{n}||y^{n-1}), \end{array}$$

where (a) follows because conditioning reduces entropy [19]; (b) follows because of the non-negativity of the discrete entropy [19]; (c) follows by definition.

Next, we prove **(ii)**. This can be shown as follows:

(19) $$\begin{array}{cc} I\left(x^{t};\ell_{t}|\ell^{t-1},y^{t-1}\right)-I\left(x^{t};y_{t}|y^{t-1}\right) & \overset{\left(d\right)}{=}I\left(x^{t};\ell_{t},y_{t}|\ell^{t-1},y^{t-1}\right)-I\left(x^{t};y_{t}|y^{t-1}\right)\\ \; & \overset{\left(e\right)}{=}I\left(x^{t};\ell^{t}|y^{t}\right)-I\left(x^{t};\ell^{t-1}|y^{t-1}\right),\\ \; & \overset{\left(f\right)}{=}I\left(x^{t};\ell^{t}|y^{t}\right)-I\left(x^{t-1};\ell^{t-1}|y^{t-1}\right), \end{array}$$

where (d) follows from an adaptation of ([20], Lemma 3.3) to processes, i.e., $I\left(x^{t};\ell_{t},y_{t}|\ell^{t-1},y^{t-1}\right)=I\left(x^{t};\ell_{t}|\ell^{t-1},y^{t-1}\right)+I\left(x^{t};y_{t}|\ell^{t},y^{t-1}\right)$ and the second term is zero because of the conditional independence constraint (16); (e) follows by the chain rule of conditional mutual information (again an adaptation of ([20], Lemma 3.3)) which decomposes the conditional mutual information in two different ways, i.e.,

$$\begin{array}{c} \begin{array}{cc} I(x^{t};\ell^{t},y_{t}|y^{t-1}) & =I\left(x^{t};\ell^{t-1}|y^{t-1}\right)+I\left(x^{t};\ell_{t},y_{t}|\ell^{t-1},y^{t-1}\right)\\ \; & =I\left(x^{t};\ell^{t}|y^{t}\right)+I\left(x^{t};y_{t}|y^{t-1}\right); \end{array} \end{array}$$

(f) follows because an adaptation of ([20], Lemma 3.3) can be applied to $I(x^{t};\ell^{t-1}|y^{t-1})$ as follows

$$\begin{array}{c} \begin{array}{cc} I(x^{t};\ell^{t-1}|y^{t-1}) & =I(x_{t},x^{t-1};\ell^{t-1}|y^{t-1})\\ \; & =I\left(x_{t};\ell^{t-1}|x^{t-1},y^{t-1}\right)+I\left(x^{t-1};\ell^{t-t}|y^{t-1}\right)\\ \; & \overset{\left(g\right)}{=}I\left(x^{t-1};\ell^{t-t}|y^{t-1}\right),\;\forall t, \end{array} \end{array}$$

where (g) follows because $I(x_{t};\ell^{t-1}|x^{t-1},y^{t-1})=0$. This can be shown as follows.

(20) $$\begin{array}{cc} I(x_{t};\ell^{t-1}|x^{t-1},y^{t-1}) & =h\left(x_{t}|x^{t-1},y^{t-1}\right)-h\left(x_{t}|x^{t-1},y^{t-1},\ell^{t-1}\right)\\ \; & =h\left(x_{t}|x^{t-1},y^{t-1}\right)-h\left(x_{t}|x^{t-1}\right)\leq 0,\;\forall t, \end{array}$$

where each h(·) is assumed to be finite for any *t*, and (20) follows from the conditional independence constraint in (15). From the non-negativity of conditional mutual information [19], the result follows.

Finally, from (19) we have

(21) $$\begin{array}{cc} \sum_{t=0}^{n}\left[I\left(x^{t};\ell^{t}|y^{t}\right)-I\left(x^{t-1};\ell^{t-1}|y^{t-1}\right)\right] & =I(x_{0};\ell_{0}|y_{0})+I\left(x^{1};\ell^{1}|y^{1}\right)-I\left(x_{0};\ell_{0}|y_{0}\right)+\ldots \\ \; & \;\ldots +I\left(x^{n};\ell^{n}|y^{n}\right)-I\left(x^{n-1};\ell^{n-1}|y^{n-1}\right) \end{array}$$

(22) $$\begin{array}{cc} \; & =I(x^{n};\ell^{n}|y^{n})\geq 0, \end{array}$$

where (22) follows by applying the method of differences in (21). The result follows because (22) is by definition non-negative. We note that if $I(x_{0};\ell_{0}|y_{0})$ also appeared in the cancellations, then, this would have been the telescopic sum of the series. This completes the derivation. □

We note that Theorem 1 is different from the data processing theorem derived in ([21], Lemma 1) in that we assume the conditional independence constraint (15) instead of the conditional independence constraint $P\left(dx_{t}|x^{t-1},y^{t-1},\ell^{t-1}\right)=P\left(dx_{t}|x^{t-1},y^{t-1}\right)-a.s.$, i.e., the source process is not allowed to have access via feedback to the previous output symbols $y^{t-1}$. This technical difference results into having the mutual information in (18) subject to conditional independence constraints, instead of the well-known directed information [22].

Before we introduce the information theoretic definitions that correspond to lower bounds on (11)–(13), we formally show the construction of $I(x^{n};y^{n})$.

**Source.** The augmented source process $\left\{x_{t}:\;t\in N_{0}\right\}$ induces the sequence of conditional distributions $\left\{P\left(dx_{t}|x^{t-1}\right),\;t\in N_{0}^{n}\right\}$. Since no initial information is assumed, the distribution at t=0 is $P(dx_{0})$. In addition, by Bayes’ rule we obtain $P\left(dx^{n}\right)≜{⊗}_{t=0}^{n}P\left(dx_{t}|x^{t-1}\right)$.

**Reproduction or “test-channel”.** The reproduction process $\left\{y_{t}:\;t\in N_{0}^{n}\right\}$ parameterized by $X^{t}$ induces the sequence of conditional distributions, known as test-channels, by $\left\{P\left(dy_{t}|y^{t-1},x^{t}\right),\;t\in N_{0}^{n}\right\}$. At t=0, no initial state information is assumed, hence $P\left(dy_{0}|y^{-1},x^{0}\right)=P\left(dy_{0}|x_{0}\right)$. In addition, by Bayes’ rule we obtain $\vec{Q}\left(dy^{n}|x^{n}\right)≜{⊗}_{t=0}^{n}P\left(dy_{t}|y^{t-1},x^{t}\right)$.

From ([23], Remark 1), it can be shown that the sequence of conditional distributions $\left\{P\left(dx_{t}|x^{t-1}\right):\;t\in N_{0}^{n}\right\}$ and $\left\{P\left(dy_{t}|y^{t-1},x^{t}\right):\;t\in N_{0}^{n}\right\}$ uniquely define the family of conditional distributions on $X^{n}$ and $Y^{n}$ parameterized by $x^{n}\in X^{n}$, respectively, given by the joint distribution

(23) $$\begin{array}{c} P\left(dx^{n},dy^{n}\right)=P\left(dx^{n}\right)⊗\vec{Q}\left(dy^{n}|x^{n}\right). \end{array}$$

In addition, from (23), we can uniquely define the $Y^{n}—$marginal distribution by

$$\begin{array}{c} P\left(dy^{n}\right)≜\int_{X^{n}}P\left(dx^{n}\right)⊗\vec{Q}\left(dy^{n}|x^{n}\right), \end{array}$$

and the conditional distributions $\left\{P\left(dy_{t}|y^{t-1}\right):\;t\in N_{0}^{n}\right\}$.

Given the above construction of distributions, we can formally introduce the information measure using relative entropy as follows:

(24) $$\begin{array}{c} \begin{array}{cc} I(x^{n};y^{n}) & \overset{\left(a\right)}{≜}D(P\left(dx^{n},dy^{n}\right)||P\left(dx^{n}\right)\times P\left(dy^{n}\right))\in \left[0,\infty \right]\\ \; & \overset{\left(b\right)}{=}\int_{X^{n}\times Y^{n}}\log\left(\frac{d\vec{Q}\left(\cdot |x^{n}\right)}{dP(\cdot )}\left(y^{n}\right)\right)P\left(dx^{n},dy^{n}\right)\\ \; & \overset{\left(c\right)}{=}\sum_{t=0}^{n}E\left\{\log\left(\frac{dP(\cdot |y^{t-1},x^{t})}{dP(\cdot |y^{t-1})}\left(y_{t}\right)\right)\right\}\\ \; & \overset{\left(d\right)}{=}\sum_{t=0}^{n}I\left(x^{t};y_{t}|y^{t-1}\right), \end{array} \end{array}$$

where (a) follows by definition of relative entropy between $P(dx^{n},dy^{n})$ and the product distribution $P\left(dx^{n}\right)\times P\left(dy^{n}\right)$; (b) is due to the Radon–Nikodym derivative theorem ([23], Appendix A and Appendix C); (c) is due to chain rule of relative entropy; (d) follows by definition.

We now state as a definition the lower bounds on (11)–(13).

**Definition** **4**  (Lower bounds). *Using the previous construction of distributions and the information measure of (24), we can define the following lower bounds on (11)–(13).*

**(1)**  *The sum-rate subject to per-dimension distortion constraint is defined as follows:*
  (25) $$\begin{array}{c} R_{\mathrm{pd}}^{LB}\left(D\right)≜\underset{\begin{array}{c} P(dy_{t}|y^{t-1},x^{t}):\;t=0,\ldots ,\infty \\ \;\Sigma_{\Delta_{ii}}\leq D_{ii},\;i=1,\ldots ,p \end{array}}{\inf}\underset{n⟶\infty }{lim sup}\frac{1}{n+1}I\left(x^{n};y^{n}\right), \end{array}$$
  *with*
  (26) $$\begin{array}{c} R_{\mathrm{pd},[0,n]}^{LB}\left(D\right)≜\underset{\begin{array}{c} P\left(dy_{t}|y^{t-1},x^{t}\right):\;t\in N_{0}^{n}\\ \;\Sigma_{\Delta_{ii},t}\leq D_{ii},\;i=1,\ldots ,p \end{array}}{\inf}I\left(x^{n};y^{n}\right). \end{array}$$
  *where $\Sigma_{\Delta ,t}≜\frac{1}{n+1}\sum_{t=0}^{n}E\left\{\left(x_{t}-y_{t}\right){\left(x_{t}-y_{t}\right)}^{T}\right\}$, $\Sigma_{\Delta_{ii},t}≜\frac{1}{n+1}\sum_{t=0}^{n}{\left[E\{\left(x_{t}-y_{t}\right){\left(x_{t}-y_{t}\right)}^{T}\}\right]}_{ii}$ and D≥0.*
**(2)**  *The sum-rate subject to joint distortion constraints is defined as follows:*
  (27) $$\begin{array}{c} R_{\mathrm{joint}}^{LB}\left(D^{*}\right)≜\underset{\begin{array}{c} P(dy_{t}|y^{t-1},x^{t}):\;t=0,\ldots ,\infty \\ \;\Sigma_{\Delta_{ii}}\leq D_{ii},\;i=1,\ldots ,p\\ {lim sup}_{n⟶\infty }\frac{1}{n+1}\sum_{t=0}^{n}E\left\{\left|\right|x_{t}-y_{t}{\left|\right|}_{2}^{2}\right\}\leq D_{{}^{T}} \end{array}}{\inf}\underset{n⟶\infty }{lim sup}\frac{1}{n+1}I\left(x^{n};y^{n}\right), \end{array}$$
  (28) $$\begin{array}{c} R_{\mathrm{joint},[0,n]}^{LB}\left(D^{*}\right)≜\underset{\begin{array}{c} P\left(dy_{t}|y^{t-1},x^{t}\right):\;t\in N_{0}^{n}\\ \;\Sigma_{\Delta_{ii},t}\leq D_{ii},\;i=1,\ldots ,p\\ \frac{1}{n+1}\sum_{t=0}^{n}E\left\{\left|\right|x_{t}-y_{t}{\left|\right|}_{2}^{2}\right\}\leq D_{{}^{T}} \end{array}}{\inf}I\left(x^{n};y^{n}\right), \end{array}$$
  where $D^{*}=\min\left\{D,D_{{}^{T}}\right\}$.
**(3)**  *The sum-rate subject to covariance matrix distortion constraints is defined as follows:*
  (29) $$\begin{array}{c} R^{LB}\left(D_{\mathrm{cov}}\right)≜\underset{\begin{array}{c} P(dy_{t}|y^{t-1},x^{t}):\;t=0,\ldots ,\infty \\ \;\Sigma_{\Delta }⪯D_{\mathrm{cov}} \end{array}}{\inf}\underset{n⟶\infty }{lim sup}\frac{1}{n+1}I\left(x^{n};y^{n}\right), \end{array}$$
  (30) $$\begin{array}{c} R_{\left[0,n\right]}^{LB}\left(D_{\mathrm{cov}}\right)≜\underset{\begin{array}{c} P\left(dy_{t}|y^{t-1},x^{t}\right):\;t\in N_{0}^{n}\\ \;\Sigma_{\Delta ,t}⪯D_{\mathrm{cov}} \end{array}}{\inf}I\left(x^{n};y^{n}\right), \end{array}$$
  *where $D_{\mathrm{cov}}⪰0$.*

Next, we stress some technical remarks related to the new information theoretic measures in Definition 4 that can be obtained using known results in the literature and some known lower bounds that use the same objective function with (26)–(30).

**Remark** **2**  (Comments on Definition 4). *It can be shown that the infimization problems (26), (28) and (30), in contrast to their operational counterparts (11)–(13) are convex with respect to their test channel. This can be shown following, for instance, the techniques of [23]. By the structural properties of the test channel derived in ([24], Section 4), if the source is first-order Markov, i.e., with distribution $P\left(dx_{t}|x_{t-1}\right),\;t\in N_{0}^{n}$, the test channel distribution is of the form $P\left(dy_{t}|y^{t-1},x_{t}\right),\;t\in N_{0}^{n}$. Finally, combining this structural result, with ([25], Theorem 1.8.6), it can be shown that if $x^{n}$ is Gaussian then a jointly Gaussian process $\left\{\left(x_{t},y_{t}\right):\;t\in N_{0}\right\}$ achieves a smaller value of the information rates, and if $x^{n}$ is Gaussian and Markov, then the infimum in (26), (28) and (30) can be restricted to test channel distributions which are Gaussian, of the form $P(dy_{t}|y_{t-1},x_{t})$.*

*We recall that when the distortion constraint set contains only (9), its finite time horizon counterpart or the per instant distortion constraint $E\{||x_{t}-y_{t}{\left|\right|}_{2}^{2}\}\leq D_{t}\;\forall t$, we end up having the well known nonanticipatory-ϵ entropy [26] also found in the literature as sequential or nonanticipative RDF [27,28]. Nonanticipatory-ϵ entropy received significant interest during the last twenty years in an anthology of papers (see, e.g., [11,16,24,29,30,31]) due to its utility in control related and delay-constrained applications. Moreover, the characterizations in (29) and (30) do not appear to be manageable to solve using standard techniques, and no closed-form statements are available for the general RDF in the literature. For this reason, we will seek only for non-tight bounds.*

In view of the above, in the sequel we characterize and compute lower bounds on Definitions 1–3 for Gauss–Markov processes and for Markov models driven by additive i.i.d. noise processes.

### 2.1. Characterization and Computation of Jointly Gaussian Processes

In this section, we assume that the augmented joint process $\left\{\left(x_{t},y_{t}\right):\;t\in N_{0}\right\}$ is jointly Gaussian. We use this assumption to first characterize and then to compute optimally (26), (28) and (30).

We first use the following helpful lemma. We exclude the proof because it is already derived in other papers, see, e.g., [14,24]. The only modification is the augmented joint processes $\left\{\left(x_{t},\;y_{t}\right):\;t\in N_{0}^{n}\right\}$.

**Lemma** **1**  (Realization of $\left\{P^{*}\left(dy_{t}|y^{t-1},x_{t}\right):\;t\in N_{0}^{n}\right\}$). Consider the class of conditionally Gaussian test channels $\left\{P^{*}\left(dy_{t}|y^{t-1},x_{t}\right):\;t\in N_{0}^{n}\right\}$. Then, the following statements hold.

**(1)**  Any candidate of $\left\{P^{*}\left(dy_{t}|y^{t-1},x_{t}\right):\;t\in N_{0}^{n}\right\}$ can be realized by the recursion
  (31) $$\begin{array}{cc} y_{t}= & H_{t}\left(x_{t}-{\hat{x}}_{t|t-1}\right)+{\hat{x}}_{t|t-1}+v_{t},\;{\hat{x}}_{0|-1}=given,\;t\in N_{0}^{n}, \end{array}$$
  *where ${\hat{x}}_{t|t-1}≜E\left\{x_{t}|y^{t-1}\right\}$, $\left\{v_{t}\in R^{p_{1}+p_{2}}∼N\left(0;\Sigma_{v_{t}}\right):\;t\in N_{0}^{n}\right\}$ is an independent Gaussian process independent of $\left\{w_{t}:\;t\in N_{0}^{n-1}\right\}$ and $x_{0}$, and $\left\{H_{t}\in R^{\left(p_{1}+p_{2}\right)\times \left(p_{1}+p_{2}\right)}:\;\;t\in N_{0}^{n}\right\}$ are time-varying deterministic matrices.*
  *Moreover, the innovations process $\left\{I_{t}\in R^{p_{1}+p_{2}}:\;t\in N_{0}^{n}\right\}$ of (31) is the orthogonal process defined by*
  $$\begin{array}{c} I_{t}≜y_{t}-E\left\{y_{t}|y^{t-1}\right\}=H_{t}\left(x_{t}-{\hat{x}}_{t|t-1}\right)+v_{t}, \end{array}$$
  *where $I_{t}∼N\left(0;\Sigma_{I_{t}}\right)$, $\Sigma_{I_{t}}=H_{t}\Sigma_{t|t-1}H_{t}^{T}+\Sigma_{v_{t}}$ and $\Sigma_{t|t-1}≜E\left\{\left(x_{t}-{\hat{x}}_{t|t-1}\right){\left(x_{t}-{\hat{x}}_{t|t-1}\right)}^{T}|y^{t-1}\right\}=E\left\{\left(x_{t}-{\hat{x}}_{t|t-1}\right){\left(x_{t}-{\hat{x}}_{t|t-1}\right)}^{T}\right\}$.*
**(2)**  *Let ${\hat{x}}_{t|t}≜E\left\{x_{t}|y^{t}\right\}$ and $\Sigma_{t|t}≜E\left\{\left(x_{t}-{\hat{x}}_{t|t}\right){\left(x_{t}-{\hat{x}}_{t|t}\right)}^{T}|y^{t}\right\}=E\left\{\left(x_{t}-{\hat{x}}_{t|t}\right){\left(x_{t}-{\hat{x}}_{t|t}\right)}^{T}\right\}$. Then, $\left\{{\hat{x}}_{t|t-1},\;\Sigma_{t|t-1}:t\in N_{0}^{n}\right\}$ satisfy the following vector-valued equations:*
  (32) $$\begin{array}{c} \begin{array}{cc} \; & {\hat{x}}_{t|t-1}=A{\hat{x}}_{t-1|t-1},\\ \; & \Sigma_{t|t-1}=A\Sigma_{t-1|t-1}A^{T}+\Sigma_{w_{t}},\\ \; & {\hat{x}}_{t|t}={\hat{x}}_{t|t-1}+N_{t}I_{t},\\ \; & N_{t}=\Sigma_{t|t-1}H_{t}^{T}\Sigma_{I_{t}}^{-1}\;\left(Kalman\;Gain\right),\\ \; & \Sigma_{t|t}=\Sigma_{t|t-1}-\Sigma_{t|t-1}H_{t}^{T}\Sigma_{I_{t}}^{-1}H_{t}\Sigma_{t|t-1}, \end{array} \end{array}$$
  *where $\Sigma_{t|t}⪰0$ and $\Sigma_{t|t-1}≻0$.*
**(3)**  Using MMSE estimation via the vector-valued KF recursions of (32), the following finite dimensional characterizations of $R_{\mathrm{pd},[0,n]}^{LB,G}\left(D\right),\;R_{\mathrm{joint},[0,n]}^{LB,G}\left(D^{*}\right),\;R_{\mathrm{cov},[0,n]}^{LB,G}\left(D_{\mathrm{cov}}\right)$ can be obtained:
  (33) $$\begin{array}{cc} R_{\mathrm{pd},[0,n]}^{LB,G}\left(D\right) & =\underset{\begin{array}{c} H_{t}\in R^{\left(p_{1}+p_{2}\right)\times \left(p_{1}+p_{2}\right)},\;\Sigma_{v_{t}}⪰0,\;t\in N_{0}^{n}\\ 0\leq {\tilde{\Sigma }}_{ii,t}\leq D_{ii},\;i=1,\ldots ,p \end{array}}{\inf}\frac{1}{2}\sum_{t=0}^{n}{\left[\log\frac{|\Sigma_{t|t-1}|}{|\Sigma_{t|t}|}\right]}^{+}, \end{array}$$
  (34) $$\begin{array}{cc} R_{\mathrm{joint},[0,n]}^{LB,G}\left(D^{*}\right) & =\underset{\begin{array}{c} H_{t}\in R^{\left(p_{1}+p_{2}\right)\times \left(p_{1}+p_{2}\right)},\;\Sigma_{v_{t}}⪰0,\;t\in N_{0}^{n}\\ 0\leq {\tilde{\Sigma }}_{ii,t}\leq D_{ii},\;i=1,\ldots ,p\\ \frac{1}{n+1}\sum_{t=0}^{n}\mathrm{trace}\left(\left(I_{p_{1}+p_{2}}-H_{t}\right)\Sigma_{t|t-1}{\left(I_{p_{1}+p_{2}}-H_{t}\right)}^{T}+\Sigma_{v_{t}}\right)\leq D_{{}^{T}} \end{array}}{\inf}\frac{1}{2}\sum_{t=0}^{n}{\left[\log\frac{|\Sigma_{t|t-1}|}{|\Sigma_{t|t}|}\right]}^{+}, \end{array}$$
  (35) $$\begin{array}{cc} R_{\left[0,n\right]}^{LB,G}\left(D_{\mathrm{cov}}\right) & =\underset{\begin{array}{c} H_{t}\in R^{\left(p_{1}+p_{2}\right)\times \left(p_{1}+p_{2}\right)},\;\Sigma_{v_{t}}⪰0,\;t\in N_{0}^{n}\\ \frac{1}{n+1}\sum_{t=0}^{n}\left(\left(I_{p_{1}+p_{2}}-H_{t}\right)\Sigma_{t|t-1}{\left(I_{p_{1}+p_{2}}-H_{t}\right)}^{T}+\Sigma_{v_{t}}\right)⪯D_{\mathrm{cov}} \end{array}}{\inf}\frac{1}{2}\sum_{t=0}^{n}{\left[\log\frac{|\Sigma_{t|t-1}|}{|\Sigma_{t|t}|}\right]}^{+}, \end{array}$$
  where ${\tilde{\Sigma }}_{ii,t}≜\frac{1}{n+1}\sum_{t=0}^{n}{\left[\left(I_{p_{1}+p_{2}}-H_{t}\right)\Sigma_{t|t-1}{\left(I_{p_{1}+p_{2}}-H_{t}\right)}^{T}+\Sigma_{v_{t}}\right]}_{ii}\geq 0$, $D\in [0,D_{\max}]$ and $D^{*}\in \left[0,D_{\max}^{*}\right]$.

We note that one can directly study the finite-dimensional characterizations of Lemma 1, **(3)**, and try to come up with numerical solutions. However, it is much more insightful to use instead the identification of the design parameters $\left\{\left(H_{t},\;\Sigma_{v_{t}}\right):\;t\in N_{0}^{n}\right\}$ of the test-channel realization in (31). This approach is already done in [14,24] hence we state it without a proof. Note, however, that compared to [14,24] that assume distortion constraints like (9) (or the per time instant counterpart of (9), i.e., $E\left\{\left|\right|x_{t}-y_{t}{\left|\right|}_{2}^{2}\right\}\leq D_{t},\;\forall t$), here we assume augmented state-space models and various spatio-temporal distortion constraints, namely, per-dimension, jointly per-dimension and averaged total distortion constraints, and covariance matrix distortion constraint.

**Lemma** **2**  (Alternative characterizations of (33)–(35) via system identification). *Consider Lemma 1 and set $\Delta_{t}≜\Sigma_{t|t}$ and $\Lambda_{t}≜\Sigma_{t|t-1}$. Then, the following statements hold.*

**(1)**  *The test-channel distribution $P(dy_{t}|y^{t-1},x_{t})$ admits the following linear Markov additive noise realization:*
  (36) $$\begin{array}{c} y_{t}=H_{t}x_{t}+\left(I_{p_{1}+p_{2}}-H_{t}\right)Ay_{t-1}+v_{t},\;y_{-1}=given,\;t\in N_{0}^{n}, \end{array}$$
  *where*
  (37) $$\begin{array}{c} H_{t}≜I_{p_{1}+p_{2}}-\Delta_{t}\Lambda_{t}^{-1},\;\Sigma_{v_{t}}≜\Delta_{t}H_{t}^{T}⪰0. \end{array}$$
**(2)**  The finite-dimensional characterizations of $R_{\mathrm{pd},[0,n]}^{LB,G}\left(D\right),\;R_{\mathrm{joint},[0,n]}^{LB,G}\left(D^{*}\right),\;R_{\mathrm{cov},[0,n]}^{LB,G}\left(D_{\mathrm{cov}}\right)$ can be simplified to the following alternative characterizations:
  (38) $$\begin{array}{cc} R_{\mathrm{pd},[0,n]}^{LB,G}\left(D\right) & =\underset{0\leq \Delta_{ii,t}\leq D_{ii},\;i=1,\ldots ,p,\;t\in N_{0}^{n}}{\inf}\frac{1}{2}\sum_{t=0}^{n}{\left[\log\frac{|\Lambda_{t}|}{|\Delta_{t}|}\right]}^{+}, \end{array}$$
  (39) $$\begin{array}{cc} R_{\mathrm{joint},[0,n]}^{LB,G}\left(D^{*}\right) & =\underset{\begin{array}{c} 0\leq \Delta_{ii,t}\leq D_{ii},\;i=1,\ldots ,p,\;t\in N_{0}^{n},\\ \frac{1}{n+1}\sum_{t=0}^{n}\mathrm{trace}\left(\Delta_{t}\right)\leq D_{{}^{T}} \end{array}}{\inf}\frac{1}{2}\sum_{t=0}^{n}{\left[\log\frac{|\Lambda_{t}|}{|\Delta_{t}|}\right]}^{+}, \end{array}$$
  (40) $$\begin{array}{cc} R_{\left[0,n\right]}^{LB,G}\left(D_{\mathrm{cov}}\right) & =\underset{\frac{1}{n+1}\sum_{t=0}^{n}\left(\Delta_{t}\right)⪯D_{\mathrm{cov}}}{\inf}\frac{1}{2}\sum_{t=0}^{n}{\left[\log\frac{|\Lambda_{t}|}{|\Delta_{t}|}\right]}^{+}, \end{array}$$
  *where $\Delta_{ii,t}$ is defined precisely as ${\tilde{\Sigma }}_{ii,t}$.*

Next, we give some technical remarks related to Lemma 2.

**Remark** **3**  (Special case and technical remarks).

**(1)**  *Clearly, if in the forward test-channel realization with additive noise, we assume that the block diagonal matrix A=0 (null matrix), then, we recover the classical forward test-channel realization for vector memoryless Gaussian source subject to a MSE distortion (see, e.g., ([32], Chapter 4.5), ([15], Chapter 9.7)) given by*
  (41) $$\begin{array}{c} y_{t}=H_{t}x_{t}+v_{t},\;t\in N_{0}^{n}, \end{array}$$
  *and the coefficients in (37) give*
  (42) $$\begin{array}{c} H_{t}≜I_{p_{1}+p_{2}}-\Delta_{t}\Sigma_{w}^{-1}⪰0,\;\Sigma_{v_{t}}≜\Delta_{t}H_{t}^{T}⪰0. \end{array}$$
  *Moreover, the characterizations in (38)–(40) change in that $\Lambda_{t}=\Sigma_{w}$. Clearly, (42) can be seen as reverse-waterfilling design parameters.*
**(2)**  *Compared to***(1)***, we note that $H_{t}$ in (37) should not be confused with a positive semidefinite matrix defined in the usual quadratic form [33] but instead it can possibly be a non-symmetric matrix which however contains only real non-negative eigenvalues. This observation is important because it means that in general the design variables $\left(\Delta_{t},\Lambda_{t}\right)$ do not commute like in the classical reverse-waterfilling problems for memoryless multivariate Gaussian random variables or in i.i.d. processes (see, e.g., [19,25]).*
**(3)**  For jointly Gaussian processes, the linear forward realization in (36) is the optimal realization among all realizations for this problem because the KF is the optimal causal MMSE estimator. Beyond Gaussian processes, and when the noise is zero-mean, uncorrelated and white (in our setup these properties hold), the optimal realization for Gaussian processes becomes the best linear realization (see, e.g., ([34], §3.2) or ([35], p. 130)) and similarly the corresponding characterizations in (38)–(40) are the best linear characterizations. By saying “best-linear” realization and characterizations, respectively, we mean that there may be non-linear realizations and hence non-linear-based characterizations that outperform the best linear.
**(4)**  The characterization of (39) is different from the characterization obtained in ([16], Theorem 1, (25e)) that uses weighted distortion constraints. The former optimization problem imposes hard constraints whereas the latter imposes soft constraints via weights. Nonetheless, an interesting open question is whether there exists a set of weights, which will give the same per dimension distortion when imposed as a weighted total distortion constraint.
**(5)**  It should also be stressed that the per-dimension constraints on the diagonal entries of $\Delta_{t}$ are not the same as having constraints on the eigenvalues of $\Delta_{t}$. This further means that even for this class of distortion constraints, it is still possible to have rate-distortion resource allocation (i.e., a type of reverse-waterfilling optimization).

**Remark** **4**  (Convexity). *The optimization problems in (38) and (39) are convex because the objective function is linear and the constraints are affine and positive semi-definite. Thus, the problem can be solved numerically using convex programming software (see, e.g, [36]) or the more challenging KKT conditions that are first-order necessary conditions for global optimality ([37], Chapter 5.3). The latter, will give certain non-linear matrix Riccati equations that need to be solved in order to construct a reverse-waterfilling algorithm.*

**Remark** **5**  (Existence of Solution). *A sufficient condition for existence of a solution with a finite value in (38)–(40) is to consider the strict LMI constraint $0≺\Delta_{t}⪯\Lambda_{t}$ that ensures the objective function is bounded. The strict LMI ensures that $\Delta_{t}≻0$ which further means that D>0, $D_{{}^{T}}>0$ and $D_{\mathrm{cov}}≻0$.*

In what follows, we derive lower bounds on (11)–(13).

**Lemma** **3**  (Steady-state lower bounds on (11)–(13)). *Suppose that the conditions of Remark 5 hold. Moreover, let $\Delta ≜\frac{1}{n+1}\sum_{t=0}^{n}\Delta_{t}$ for some finite n. Then, the following statements hold.*

**(1)**  (43) $$\begin{array}{c} R_{\mathrm{pd}}^{c}\left(D\right)\geq \min_{\begin{array}{c} 0≺\Delta ⪯\Lambda \\ \Delta_{ii}\leq D_{ii},\;i=1,\ldots ,p \end{array}}\frac{1}{2}\log\frac{\left|\Lambda \right|}{\left|\Delta \right|}, \end{array}$$
  *where $\Lambda =A\Delta A^{T}+\Sigma_{w}$.*
**(2)**  (44) $$\begin{array}{c} R_{\mathrm{joint}}^{c}\left(D^{*}\right)\geq \min_{\begin{array}{c} 0≺\Delta ⪯\Lambda \\ \Delta_{ii}\leq D_{ii},\;i=1,\ldots ,p\\ \mathrm{trace}(\Delta )\leq D \end{array}}\frac{1}{2}\log\frac{\left|\Lambda \right|}{\left|\Delta \right|}, \end{array}$$
  for some $D^{*}=\min\left\{D_{{}^{T}},D\right\}$.
**(3)**  (45) $$\begin{array}{c} R^{c}\left(D_{\mathrm{cov}}\right)\geq \min_{\begin{array}{c} 0≺\Delta ⪯\Lambda \\ 0≺\Delta ⪯D_{\mathrm{cov}} \end{array}}\frac{1}{2}\log\frac{\left|\Lambda \right|}{\left|\Delta \right|}. \end{array}$$

**Proof.**  See Appendix A. □

It should be remarked that instead of the derivation based on a convexity argument in Lemma 3, one can assume that the optimal minimizer $P(dy_{t}|y_{t-1},x_{t})$ that achieves (43)–(45) is time-invariant and the output distribution $P(dy_{t}|y_{t-1})$ is also time-invariant with a unique invariant distribution, see, e.g., ([14], Theorem 3). Moreover, the optimal linear forward test-channel that achieves the lower bounds in (43)–(45) correspond to the time-invariant realization (36), given by

(46) $$\begin{array}{c} y_{t}=Hx_{t}+\left(I_{p_{1}+p_{2}}-H\right)Ay_{t-1}+v_{t}, \end{array}$$

whereas the corresponding time-invariant scaling coefficients in (37) are as follows

(47) $$\begin{array}{c} H≜I_{p_{1}+p_{2}}-\Delta \Lambda^{-1},\;\Sigma_{v}≜\Delta H^{T}⪰0. \end{array}$$

From Lemma 3, the following corollary can be immediately obtained.

**Corollary** **1**  (Fixed design variable Δ). *If in Lemma 2 we assume that $\Delta_{t}=\Delta$, ∀t, then we obtain (43)–(45).*

**Proof.**  This is immediate from the derivation of Lemma 3. □

In what follows, we show that the lower bounds in Lemma 3 are semidefinite representable, thus, they can be readily computed.

**Theorem** **2**  (Computation of the lower bounds in Lemma 3). *Consider the variable $Q_{1}≜\Delta^{-1}-A{}^{T}\Sigma_{w}^{-1}A$, where Δ≻0. Then, the following semidefinite programming representations hold.*

**(1)**  *For some $D\equiv \mathrm{trace}(\hat{D})>0$, the lower bound in (43), denoted hereinafter by $R_{\mathrm{pd}}^{LB}\left(D\right)$, is semidefinite representable as follows:*
  (48) $$\begin{array}{cc} R_{\mathrm{pd}}^{LB}\left(D\right) & =\min_{\begin{array}{c} Q_{1}≻0 \end{array}}-\frac{1}{2}\log|Q_{1}|+\frac{1}{2}\log\left|\Sigma_{w}\right|.\\ s.t.\; & 0≺\Delta ⪯\Lambda \\ \; & \Delta_{ii}\leq D_{ii},\;i=1,\ldots ,p \end{array}$$
  (49) $$\begin{array}{cc} \; & \left[\begin{array}{cc} \Delta -Q_{1} & \Delta A{}^{T}\\ A\Delta & \Lambda \end{array}\right]⪰0 \end{array}$$
**(2)**  For some $D^{*}=\min\left\{D_{{}^{T}},D\right\}>0$, the lower bound in (44), denoted hereinafter by $R_{\mathrm{joint}}^{LB}\left(D^{*}\right)$, is semidefinite representable as follows:
  (50) $$\begin{array}{cc} R_{\mathrm{joint}}^{LB}\left(D^{*}\right) & =\min_{\begin{array}{c} Q_{1}≻0 \end{array}}-\frac{1}{2}\log|Q_{1}|+\frac{1}{2}\log\left|\Sigma_{w}\right|.\\ s.t.\; & 0≺\Delta ⪯\Lambda \\ \; & \Delta_{ii}\leq D_{ii},\;i=1,\ldots ,p\\ \; & \mathrm{trace}\left(\Delta \right)\leq D_{{}^{T}}\\ \; & \left[\begin{array}{cc} \Delta -Q_{1} & \Delta A{}^{T}\\ A\Delta & \Lambda \end{array}\right]⪰0 \end{array}$$
**(3)**  *For some $D_{\mathrm{cov}}≻0$, the lower bound in (45), denoted hereinafter by $R^{LB}\left(D_{\mathrm{cov}}\right)$, is semidefinite representable as follows:*
  (51) $$\begin{array}{cc} R^{LB}\left(D_{\mathrm{cov}}\right) & =\min_{\begin{array}{c} Q_{1}≻0 \end{array}}-\frac{1}{2}\log|Q_{1}|+\frac{1}{2}\log\left|\Sigma_{w}\right|.\\ s.t.\; & 0≺\Delta ⪯\Lambda \\ \; & \Delta ⪯D_{\mathrm{cov}}\\ \; & \left[\begin{array}{cc} \Delta -Q_{1} & \Delta A{}^{T}\\ A\Delta & \Lambda \end{array}\right]⪰0 \end{array}$$

**Proof.**  See Appendix B. □

Next, we stress some comments on the semidefinite representation of the lower bounds in Theorem 2.

**Remark** **6**  (Comments on Theorem 2).

**(1)**  We note that a similar characterization to the characterizations derived in Theorem 2 (subject to the distortion constraint (9) or the per-instant distortion constraint $E\{||x_{t}-y_{t}{\left|\right|}_{2}^{2}\}\leq D_{t},\;\forall t$, for a special case of the setup in Figure 1) is recently derived in ([16], Equation (27)). The log-determinant convex optimization problems in Theorem 2 are widely used in systems and control theories because they are able to deal efficiently with LMIs [38].
**(2)**  Recently, the efficiency of SDP algorithm in solving linear and non-linear optimization problems attracted experts from the field of information theory who noticed its usefulness in solving distributed source coding problems (see, e.g., [3,39]). Such log-determinant problems when solved using the semidefinite programming method are known to have polynomial worst-case complexity (see, e.g., [40]). In addition, for an interior point method such as the SDP approach, the most computationally expensive step is the Cholesky factorization involved in the Newton steps.
**(3)**  On the other hand, due to its complexity, the SDP approach for high dimensional systems is often time consuming whereas for very large scale systems is occasionally impossible to obtain numerical solutions. Hence, ideally one could preferably consider alternative methods to solve a problem sacrificing for instance the optimality of the SDP algorithm but gaining in scalability and reducing the complexity. The most computationally efficient way to compute such problems and, additionally, to gain some insight from the solution is via the well-known reverse-waterfilling algorithm ([19], Theorem 10.3.3), which is however very hard to construct and compute because one needs to employ and solve complicated KKT conditions [37]. Such effort was recently made for multivariate Gauss-Markov processes under per instant, averaged total and asymptotically averaged total distortion constraints in [24,41].

Next, we perform some numerical illustrations using the semidefinite representations of Theorem 2. We also compare (48) and (50), to the known expression obtained only for the asymptotically averaged total MSE distortion constraint in ([16], Equation (27)). We note that the SDP algorithm for (48)–(51) is implemented using the CVX platform [36].

**Example** **1**  (Comparison of $R_{\mathrm{joint}}^{LB}\left(D^{*}\right)$, $R_{\mathrm{pd}}^{LB}\left(D\right)$ and ([16], Equation (27))). *For the system in (1), we assume that user 1 is described by a $R^{2}$-valued time-invariant Markov source driven by i.i.d. Gaussian noise process with parameters $\left(A_{1},\Sigma_{w^{1}}\right)$:*

(52) $$\begin{array}{c} \left(A_{1},\;\Sigma_{w^{1}}\right)=\left(\left[\begin{array}{cc} 0.5 & 0.2\\ 0.3 & 0.6 \end{array}\right],\left[\begin{array}{cc} 1 & 0\\ 0 & 1 \end{array}\right]\right), \end{array}$$

*whereas, user 2 is described by a $R^{3}$-valued time-invariant Markov source driven by i.i.d. Gaussian noise process with parameters $\left(A_{2},\Sigma_{w^{2}}\right)$:*

(53) $$\begin{array}{c} \left(A_{2},\;\Sigma_{w^{2}}\right)=\left(\left[\begin{array}{ccc} 0.5 & 0.2 & 0.1\\ 0.3 & 0.6 & 0.1\\ 0.7 & 0.3 & 0.4 \end{array}\right],\left[\begin{array}{ccc} 1 & 0 & 0\\ 0 & 0.2 & 0\\ 0 & 0 & 0.5 \end{array}\right]\right). \end{array}$$

Clearly, the augmented state space model (2) generates $A=A_{1}⊕A_{2}$ and $\Sigma_{w}=\Sigma_{w}^{1}⊕\Sigma_{w}^{2}$. For this example, we assume that $D_{{}^{T}}=1.5$ and $D_{11}=0.1,D_{22}=0.01,D_{33}=0.6,\;D_{44}=0.15,\;D_{55}=0.1$, which implies that D=0.96. This means that $D^{*}=\min\left\{D_{{}^{T}},D\right\}=0.96$.

**Figure 3:**
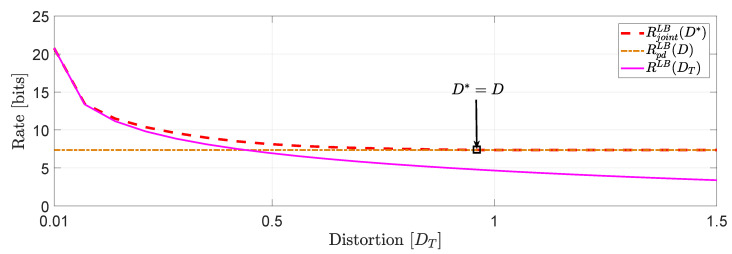
Comparison of $R_{\mathrm{joint}}^{\mathrm{na}}\left(D^{*}\right)$, $R_{\mathrm{pd}}^{\mathrm{na}}\left(D\right)$ when $D_{ii}\neq D_{jj}$, for i≠j, and comparison with ([16], Equation (27)).

In Figure 3, we compare the numerical solutions of $R_{\mathrm{joint}}^{LB}\left(D^{*}\right)$ and $R_{\mathrm{pd}}^{LB}\left(D\right)$ with ([16], Equation (27)), denoted hereinafter as $R^{LB}\left(D_{{}^{T}}\right)$.

Based on this numerical study, we observe that for distortion levels between $\left(0,D^{*}=D\right]$, $R_{\mathrm{joint}}^{LB}\left(D^{*}\right)\geq R_{\mathrm{pd}}^{LB}\left(D\right)$ whereas for values of $D_{{}^{T}}$ greater than $D^{*}$ we observe that $R_{\mathrm{joint}}^{LB}\left(D^{*}\right)=R_{\mathrm{pd}}^{LB}\left(D\right)$ because the asymptotically averaged total MSE distortion constraint is inactive. This observation verifies our comment in Section 1.2 regarding the connection of (11) and (12). Clearly, at high rates (or high resolution) we observe that $R_{\mathrm{joint}}^{LB}\left(D^{*}\right)\approx R^{LB}\left(D_{{}^{T}}\right)$.

**Figure 4:**
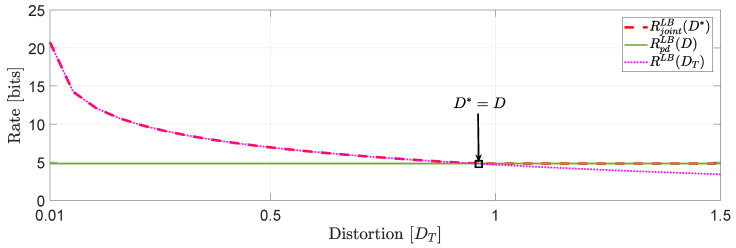
Comparison of $R_{\mathrm{joint}}^{LB}\left(D^{*}\right)$, $R_{\mathrm{pd}}^{LB}\left(D\right)$ and $R^{LB}\left(D_{{}^{T}}\right)$ when $D_{ii}=D_{jj}$, ∀i≠j.

Another interesting observation (illustrated in Figure 4) that can be made, is that if in the same example we allocate the total budget of per dimension distortion equally, i.e., $D_{ii}=D_{jj},\;\forall i\neq j$, we observe that for distortion levels between $\left(0,D^{*}=D\right]$, $R_{\mathrm{joint}}^{LB}\left(D^{*}\right)=R^{LB}\left(D_{{}^{T}}\right)\geq R_{\mathrm{pd}}^{LB}\left(D\right)$.

**Example** **2**  (Covariance matrix distortion constraint). *For the system in (1) we assume that user 1 is described by a $R^{2}$-valued time-invariant Markov source driven by i.i.d. Gaussian noise process with parameters $\left(A_{1},\Sigma_{w^{1}}\right)$:*

(54) $$\begin{array}{c} \left(A_{1},\;\Sigma_{w^{1}}\right)=\left(\left[\begin{array}{cc} 0.5 & 0.2\\ 0.3 & 0.6 \end{array}\right],\left[\begin{array}{cc} 1 & 0\\ 0 & 1 \end{array}\right]\right), \end{array}$$

*whereas, user 2 is described by an R-valued time-invariant Markov source driven by i.i.d. Gaussian noise process with parameters $\left(A_{2},\Sigma_{w^{2}}\right)$:*

(55) $$\begin{array}{c} \left(A_{2},\;\Sigma_{w^{2}}\right)=\left(0.6,2\right). \end{array}$$

*The augmented state space model (2) is generated by $A=A_{1}⊕A_{2}$ and similarly $\Sigma_{w}=\Sigma_{w}^{1}⊕\Sigma_{w}^{2}$. For this example, we assume a covariance matrix distortion constraint given by:*

(56) $$\begin{array}{c} D_{\mathrm{cov}}=\left[\begin{array}{ccc} 1.5 & \gamma & \gamma \\ \gamma & 1 & \gamma \\ \gamma & \gamma & 0.5 \end{array}\right], \end{array}$$

*where γ>0 is the positive correlation coefficient between the distortion matrix components (i.e., diagonal entries) and it is chosen such that $D_{\mathrm{cov}}≻0$.*

**Figure 5:**
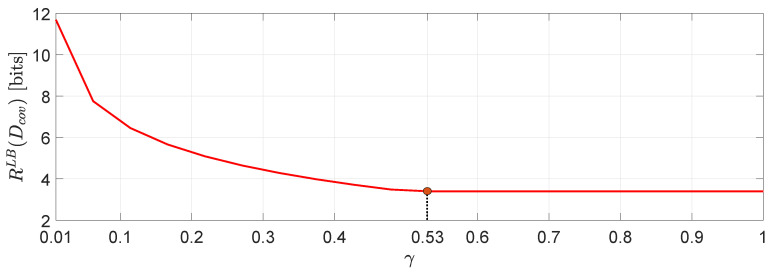
$R^{LB}\left(D_{\mathrm{cov}}\right)$ as a function of γ≥0.

*In Figure 5 we demonstrate a comparison between $R^{LB}\left(D_{\mathrm{cov}}\right)$ evaluated for several different values of γ. One interesting observation that can be made is that higher distortion correlation in (56) leads to less bits with a $\gamma_{\max}\approx 0.53$, beyond which the value of $R^{LB}\left(D_{\mathrm{cov}}\right)$ remains unchanged. Another interesting observation is that for negative correlation γ, the approximation via SDP does not give a number. However, this is not the case, in general (see, e.g., ([42], Example 1)).*

**Figure 6:**
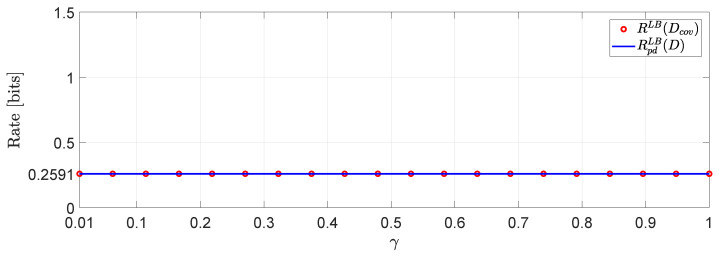
Comparison of $R^{LB}\left(D_{\mathrm{cov}}\right)$ (restricted to its values in the main diagonal) and $R_{\mathrm{pd}}^{LB}\left(D\right)$ for certain values of γ.

*Using the same simulation study, we can arrive to an interesting connection between the approximation in (51) and (48). In particular, if for instance in (56) we restrict the matrix distortion constraint only to the main diagonal elements (i.e., exactly like the the per-dimension constraints) then, we obtain the plot of Figure 6 which clearly demonstrates that $R^{LB}\left(D_{\mathrm{cov}}\right)=R_{\mathrm{pd}}^{LB}\left(D\right)$. In fact, restricting the covariance matrix distortion constraint of (56) to the per dimension distortion constraint, is as if we optimize via a solution space in which γ is allowed to have any value in R. As a result, the feasible set of solutions is larger when the constraint set is subject to per-dimension distortion constraints rather than the covariance matrix distortion constraint.*

### 2.2. Analytical Lower Bounds for Markov Sources Driven by Additive i.i.d. Noise Processes

In this subsection, we derive analytical lower bounds on (11)–(13)) when the source model describing the behavior of user 1 or user 2 is driven by possibly i.i.d. non-Gaussian noise process.

We first give a lemma which will facilitate the derivation of our lower bounds. We only consider the case of RDFs subject to per-dimension distortion constraints because the other classes of distortion constraints follow similarly.

**Lemma** **4**  (Rate-distortion bounds). *For the augmented source model describing the behavior of users 1, 2 in (3), the following inequalities hold assuming distortion constraints in the class of (8):*

(57) $$\begin{array}{c} R_{\mathrm{pd}}^{LB}\left(D\right)\overset{\left(a\right)}{\leq }R_{\mathrm{pd}}^{LB,linear}\left(D\right)\leq R_{\mathrm{pd}}^{c}\left(D\right), \end{array}$$

*where*

(58) $$\begin{array}{cc} & R_{\mathrm{pd}}^{LB,linear}\left(D\right)≜\underset{\begin{array}{c} P^{linear}\left(dy_{t}|y_{t-1},x_{t}\right):\;t\in N_{0}\\ \;\Sigma_{\Delta_{ii}}\leq D_{ii},\;i=1,\ldots ,p \end{array}}{\inf}\underset{n⟶\infty }{lim sup}\frac{1}{n+1}\sum_{t=0}^{n}I\left(x_{t};y_{t}|y_{t-1}\right), \end{array}$$

and (a) holds with equality if the augmented state space model described in (2) is jointly Gaussian and the optimal minimizer, i.e., $P^{*}\left(dy_{t}|y^{t-1},x^{t}\right)$ of $R_{\mathrm{pd}}^{LB}\left(D\right)$ is conditionally Gaussian. Equality (a) holds trivially at $D_{\max}$.

**Proof.**  The RHS inequality follows from Theorem 1 and (43) whereas the LHS inequality follows from the fact that the constraint set of $R_{\mathrm{pd}}^{LB}\left(D\right)$ is larger than the constraint set of $R_{\mathrm{pd}}^{LB,linear}\left(D\right)$ which is restricted to linear coding policies. Now, under the specific augmented source model in (3), and using Lemma 2, **(1)**, we obtain $R_{\mathrm{pd}}^{LB,linear}\left(D\right)$ defined as in (58) because these are the best linear coding policies since KF algorithm is the best linear causal MSE estimator beyond additive Gaussian noise processes (see the discussion in Remark 3, **(3)**). Clearly, if the augmented source in (3) is jointly Gaussian and the optimal realization of $R_{\mathrm{pd}}^{LB}\left(D\right)$ is conditionally Gaussian, then, the system model is jointly Gaussian and the optimal policies are linear given by the forward linear test channel realization obtained in (36) hence the LHS inequality holds with equality. □

**Remark** **7**  (Comments on Lemma 4). *We note that Lemma 4 holds if we assume RDFs with distortion constraints in the class of (9) or (10).*

The following theorem is a major result of this paper.

**Theorem** **3**  (Analytical lower bounds on (11)–(13)). *Consider the source models of users 1,2 in (1). Then, the following analytical lower bounds on (11)–(13) hold.*

**(1)**  *For $D_{ii}>0,\;\forall i$, we obtain*
  (59) $$\begin{array}{c} R_{\mathrm{pd}}^{c}\left(D\right)\geq \frac{\left(p_{1}+p_{2}\right)}{2}\log\left(|A^{T}{A|}^{\frac{1}{p_{1}+p_{2}}}+\frac{\left(p_{1}+p_{2}\right)N\left(w\right)}{D}\right), \end{array}$$
  *where $D\in \left(0,\frac{\left(p_{1}+p_{2}\right)N\left(w\right)}{1-|A^{T}{A|}^{\frac{1}{p_{1}+p_{2}}}}\right]$ with $N\left(w\right)=\frac{1}{2\pi e}2^{\frac{2}{p_{1}+p_{2}}h\left(w\right)}$ and h(w)>−∞.*
**(2)**  *For $D_{{}^{T}}>0$, and $D_{ii}>0,\;\forall i$, we obtain*
  (60) $$\begin{array}{c} R_{\mathrm{joint}}^{c}\left(D^{*}\right)\geq \frac{\left(p_{1}+p_{2}\right)}{2}\log\left(|A^{T}{A|}^{\frac{1}{p_{1}+p_{2}}}+\frac{\left(p_{1}+p_{2}\right)N\left(w\right)}{D^{*}}\right), \end{array}$$
  where $D^{*}\in \left(0,\frac{\left(p_{1}+p_{2}\right)N\left(w\right)}{1-|A^{T}{A|}^{\frac{1}{p_{1}+p_{2}}}}\right]$, with N(w) defined as in**(1)***and h(w)>−∞.*
**(3)**  *For $D_{\mathrm{cov}}≻0$ we obtain*
  (61) $$\begin{array}{c} R^{c}\left(D_{\mathrm{cov}}\right)\geq \frac{\left(p_{1}+p_{2}\right)}{2}\log\left(|A^{T}{A|}^{\frac{1}{p_{1}+p_{2}}}+\frac{N(w)}{|D_{\mathrm{cov}}{|}^{\frac{1}{p_{1}+p_{2}}}}\right), \end{array}$$
  *where $|D_{\mathrm{cov}}|\in \left(0,{\left(\frac{N(w)}{1-|A^{T}{A|}^{\frac{1}{p_{1}+p_{2}}}}\right)}^{\left(p_{1}+p_{2}\right)}\right]$, with N(w) defined as in***(1)***and h(w)>−∞.*

**Proof.**  See Appendix C. □

The following technical remarks can be made regarding Theorem 3.

**Remark** **8.** 

**(1)**  *Note that if in Theorem 3 we allow h(w)=−∞, then, the analytical lower bound expressions take a negative finite value or −∞, which cannot be the case (RDF is, by definition, non-negative). A way to include the case where h(w) is allowed to be −∞ in our lower bound expressions, is to set the objective functions in (59)–(61) to be ${\left[\log\left(\cdot \right)\right]}^{+}$. This will mean that whenever h(w)=−∞, the analytical lower bound expression will be zero.*
**(2)**  *The analytical lower bounds in (59)–(61) do not correspond to the best linear forward test channel realization of Lemma 3 (see (46)) which is also the optimal policy under the assumption of a MMSE decoder when the system’s noise is purely Gaussian (see Remark 3,* **(3)** *). Moreover, it is not clear what is the realization that achieves them the same way the bounds in Lemma 3 are achieved for Gaussian processes.*
**(3)**  *If in Theorem 3 we assume that the users 1,2 have source models described by Markov processes driven by additive Gaussian noise processes then from the EPI (see, e.g., ([43], Equation (7))) $N\left(w\right)=|\Sigma_{w}{|}^{p_{1}+p_{2}}$ and (59)–(61) change accordingly.*
**(4)**  *One can choose to further bound (61) using the inequality $|D_{\mathrm{cov}}{|}^{-\frac{1}{p_{1}+p_{2}}}\geq \frac{p_{1}+p_{2}}{\mathrm{trace}(D_{\mathrm{cov}})}$ obtaining a further lower bound that coincides with the lower bound in (59) (see also the discussion in Example 2). Such lower bound will mean that we extend the set of feasible solutions that correspond to the initial problem statement (13), to be similar to the initial problem statement of (11) which cannot be the case, in general. Our bound in (61) encapsulates the off diagonal elements of the distortion covariance matrix distortion $D_{\mathrm{cov}}$ hence it is an appropriate lower bound for the specific problem.*

In what follows, we give a numerical simulation where we compare the solution of $R_{\mathrm{joint}}^{LB}\left(D^{*}\right)$ (that corresponds to the lower bound achieved by the optimal coding policies when the system is driven by additive i.i.d. Gaussian noise processes) computed via the SDP representation of (50), with the lower bound obtained in (60) when the system’s noise is also Gaussian.

**Example** **3**  (Comparison of (50) with (60) for jointly Gaussian processes). *We consider the same input data assumed in Example 1 for users 1,2. Then, we proceed to compute the true lower bound of (50) and the lower bound obtained in (60).*

**Figure 7:**
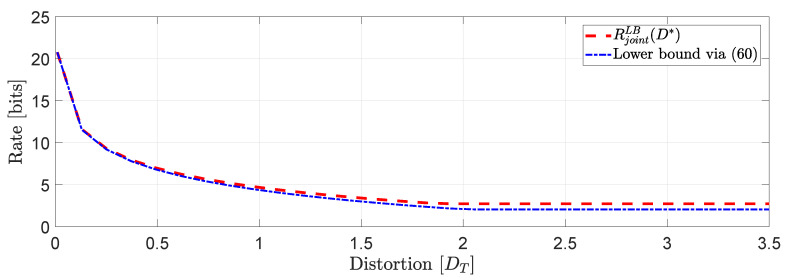
Comparison of the lower bound $R_{\mathrm{joint}}^{LB}\left(D^{*}\right)$ with the analytical expression of (60).

*Our simulation study in Figure 7 shows that at high rates the performance of the two bounds is almost identical whereas to moderate and low rates we observe a gap that remains constant when $D_{{}^{T}}\geq D$, i.e., when the asymptotically averaged total distortion constraint is inactive. The same behavior is expected for systems of larger dimension (larger scale optimization problems) with a possibility of an increased gap to moderate and low rates depending on the structure of the block diagonal matrices A and $\Sigma_{w}$.*

Next, we state a corollary of Theorem 3.

**Corollary** **2**  (Analytical bounds when users 1,2 are not specified by the same additive noise process). *Consider the source models of users 1,2 in (1). Moreover, assume that $w_{t}^{1}∼N\left(0;\Sigma_{w^{1}}\right)$ and $w_{t}^{2}∼\left(0;\Sigma_{w^{2}}\right)$ with $\Sigma_{w^{1}}⪯I_{p_{1}}$ and $\Sigma_{w^{2}}⪯I_{p_{2}}$ and $h(w^{2})>-\infty$. Then, the following analytical lower bounds on (11)–(13) hold.*

**(1)**  *For $D_{ii}>0,\;\forall i$, we obtain*
  (62) $$\begin{array}{c} R_{\mathrm{pd}}^{c}\left(D\right)\geq \frac{\left(p_{1}+p_{2}\right)}{2}\log\left(|A^{T}{A|}^{\frac{1}{p_{1}+p_{2}}}+\frac{\left(p_{1}+p_{2}\right){\left|\Sigma_{w^{1}}\right|}^{\frac{1}{p_{1}}}N\left(w^{2}\right)}{D}\right), \end{array}$$
  *where $D\in \left(0,\frac{\left(p_{1}+p_{2}\right)N\left(w\right)}{1-|A^{T}{A|}^{\frac{1}{p_{1}+p_{2}}}}\right]$ with $N\left(w^{2}\right)=\frac{1}{2\pi e}2^{\frac{2}{p_{2}}h\left(w^{2}\right)}$.*
**(2)**  *For $D_{{}^{T}}>0$, $D_{ii}>0,\;\forall i$, we obtain*
  (63) $$\begin{array}{c} R_{\mathrm{joint}}^{c}\left(D^{*}\right)\geq \frac{\left(p_{1}+p_{2}\right)}{2}\log\left(|A^{T}{A|}^{\frac{1}{p_{1}+p_{2}}}+\frac{\left(p_{1}+p_{2}\right){\left|\Sigma_{w^{1}}\right|}^{\frac{1}{p_{1}}}N\left(w^{2}\right)}{D^{*}}\right), \end{array}$$
  where $D^{*}\in \left(0,\frac{\left(p_{1}+p_{2}\right){\left|\Sigma_{w^{1}}\right|}^{\frac{1}{p_{1}}}N\left(w^{2}\right)}{1-|A^{T}{A|}^{\frac{1}{p_{1}+p_{2}}}}\right]$.
**(3)**  *For $D_{\mathrm{cov}}≻0$ we obtain*
  (64) $$\begin{array}{c} R^{c}\left(D_{\mathrm{cov}}\right)\geq \frac{\left(p_{1}+p_{2}\right)}{2}\log\left(|A^{T}{A|}^{\frac{1}{p_{1}+p_{2}}}+\frac{|\Sigma_{w^{1}}{|}^{\frac{1}{p_{1}}}N\left(w^{2}\right)}{|D_{\mathrm{cov}}{|}^{\frac{1}{p_{1}+p_{2}}}}\right), \end{array}$$
  *where $|D_{\mathrm{cov}}|\in \left(0,{\left(\frac{|\Sigma_{w^{1}}{|}^{\frac{1}{p_{1}}}N\left(w^{2}\right)}{1-|A^{T}{A|}^{\frac{1}{p_{1}+p_{2}}}}\right)}^{\left(p_{1}+p_{2}\right)}\right]$.*

**Proof.**  All cases **(1)**–**(3)** follow almost identical steps with the derivation of Theorem 3. The only different but crucial step lies in (A6) where we then use the fact that

(65) $$\begin{array}{c} |\Sigma_{w}{|}^{\frac{1}{p_{1}+p_{2}}}\overset{\left(a\right)}{=}|\Sigma_{w^{1}}{|}^{\frac{1}{p_{1}+p_{2}}}|\Sigma_{w^{2}}{|}^{\frac{1}{p_{1}+p_{2}}}\overset{\left(b\right)}{\geq }|\Sigma_{w^{1}}{|}^{\frac{1}{p_{1}}}|\Sigma_{w^{2}}{|}^{\frac{1}{p_{2}}}\overset{\left(c\right)}{\geq }{\left|\Sigma_{w^{1}}\right|}^{\frac{1}{p_{1}}}N\left(w^{2}\right), \end{array}$$

where (a) follows from properties of block diagonal matrices ([33], Section 0.9.2); (b) follows from the conditions of the corollary on the noise covariance matrices; (c) follows from the EPI ([43], Equation (7)). □

One can deduce the following for Corollary 2.

**Remark** **9.**  *Corollary 2 will give similar analytical lower bounds (with appropriate modifications) if instead of user 1, we assume that the source model of user 2 is driven by a Gaussian noise process. The additional assumption on the covariance matrix of the noise process in both users is imposed because otherwise we cannot guarantee that the key series of inequalities (65) will be satisfied.*

## 3. Upper Bounds

In this section we explain the case of encoding the augmented vector-valued Markov source modeled by (3) using a sequential causal DPCM scheme with a feedback loop followed by an ECDQ. The scheme relies on the linear forward test channel realization of the bounds in Lemma 2. The precursor of the DPCM-based scheme with feedback loop is [14] whereas ECDQ is a classical source coding approach with standard performance guarantees in information theory (see, e.g., [44]). The ECDQ scheme is utilized to bound the rate performance of the DPCM scheme. This approach furnish with an achievable (upper) bound the operation causal RDFs in (11)–(13).

### 3.1. DPCM with Feedback Loop

First, we briefly describe the sequential causal DPCM scheme with feedback loop introduced in ([14], Figure 2) (see also [45]). Observe that because the augmented source is modeled as a first-order multidimensional Markov process, the sequential causal coding is precisely equivalent to a predictive coding paradigm (see, e.g., [14,46]).

At each time instant *t*, the encoder or innovations’ encoder performs the linear operation

(66) $$\begin{array}{c} {\hat{x}}_{t}=x_{t}-Ay_{t-1}, \end{array}$$

where at t=0 we assume initial data ${\hat{x}}_{0}=x_{0}$ and also $y_{t-1}≜E\left\{x_{t-1}|\ell^{t-1}\right\}$, i.e., an estimate of $x_{t-1}$ given the previous quantized symbols $\ell^{t-1}$ (Note that the process ${\hat{x}}_{t}$ has a temporal correlation since it subtracts the error of $x_{t}$ given all previous quantized symbols $\ell^{t-1}$ and not the infinite past of the source $x_{-\infty }^{t}$. Hence, ${\hat{x}}_{t}$ is only an estimate of the true process and this causes a part of the sub-optimality of this scheme.). Then, by means of a $R^{p_{1}+p_{2}}$-valued MMSE quantizer that operates at a rate $R_{t}$, we generate the quantized reconstruction ${\hat{y}}_{t}$ of the residual source ${\hat{x}}_{t}$ denoted by ${\hat{y}}_{t}=y_{t}-Ay_{t-1}$. Then, we send $\ell_{t}$ over the channel (the corresponding data packet to ${\hat{y}}_{t}$). At the decoder we receive $\ell_{t}$ and recover the quantized symbol ${\hat{y}}_{t}$ of ${\hat{x}}_{t}$.

Then, we generate the estimate $y_{t}$ using the linear operation

(67) $$\begin{array}{c} y_{t}={\hat{y}}_{t}+Ay_{t-1}. \end{array}$$

Combining both (66) and (67), we obtain

(68) $$\begin{array}{c} x_{t}-y_{t}={\hat{x}}_{t}-{\hat{y}}_{t}. \end{array}$$

From (68), we can immediately deduce that the error between $x_{t}$ and $y_{t}$ is equal to the quantization error introduced by ${\hat{x}}_{t}$ and ${\hat{y}}_{t}$ which means that the MSE distortion at each instant of time satisfy

(69) $$\begin{array}{c} E\{||x_{t}-y_{t}{\left|\right|}_{2}^{2}\}=E\{||{\hat{x}}_{t}-{\hat{y}}_{t}{\left|\right|}_{2}^{2}\}. \end{array}$$

In addition, the covariance matrix $\Sigma_{\Delta }$ yields

(70) $$\begin{array}{c} E\left\{\left(x_{t}-y_{t}\right){\left(x_{t}-y_{t}\right)}^{T}\right\}=E\left\{\left({\hat{x}}_{t}-{\hat{y}}_{t}\right){\left({\hat{x}}_{t}-{\hat{y}}_{t}\right)}^{T}\right\}. \end{array}$$

**Figure 8:**
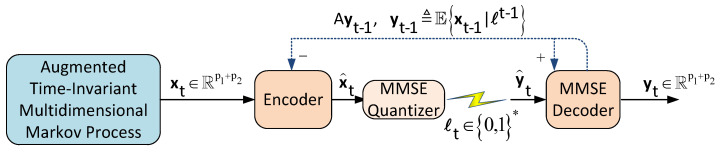
DPCM scheme with feedback loop for the augmented multidimensional Markov model of (3).

A pictorial view of the DPCM scheme with feedback loop is given in Figure 8.

### 3.2. Bounding (11)–(13) via A DPCM-based ECDQ for Gaussian Noise Processes

In this subsection, we bound the rate performance of the DPCM scheme described in Section 3.1 in the infinite time horizon, using a scheme that utilizes the steady-state linear forward test-channel realization that achieves the lower bounds of Lemma 3. Essentially, what we do in this scheme is that we replace the quantization noise with an additive Gaussian noise with the same second moments (see e.g., [47] or [44] (Chapter 5) and the references therein).

Recall that the steady-state linear forward test-channel realization of the lower bounds in Lemma 3 is written as follows:

(71) $$\begin{array}{c} y_{t}=Hx_{t}+\left(I_{p_{1}+p_{2}}-H\right)Ay_{t-1}+v_{t}, \end{array}$$

whereas the steady-state reverse-waterfilling parameters $\left(H,\;\Sigma_{v}\right)$ are given by

(72) $$\begin{array}{c} H≜I_{p_{1}+p_{2}}-\Delta \Lambda^{-1},\;\Sigma_{v}=H\Delta ⪰0. \end{array}$$

**Figure 9:**
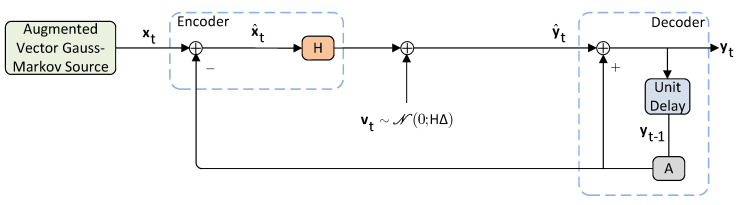
Forward test channel realization of (71).

The forward test-channel realization of (71) is illustrated in Figure 9.

Before we proceed, we point out the following important technical remarks on the realization of (71) and the coefficients (72).

**Remark** **10**  (Observations (71) and (72)). *The linear forward test channel realization with additive noise in (71) is equivalent to the steady-state realization in (46) because for both it can be shown that the MSE distortion constraint is achieved (i.e., $v_{t}∼N\left(0;\Sigma_{v}\right)$, $\Sigma_{v}=H\Delta =\Delta H^{T}⪰0$). Moreover, this realization is equivalent but simpler to build compared to the forward test channel realization introduced in [14] in which non-singular matrices and diagonalization by congruence is assumed (see ([14], Theorem 4)).*

*In the test channel realization of Figure 9, a reverse-waterfilling in spatial dimension is possible when we assume asymptotically averaged total MSE distortion constraints similar to ([14], Theorem 40). This reverse-waterfilling is dictated by the rank of matrix H. To make this point clear, if H is full rank, then all spatial dimensions in the system are active whereas if H is rank deficient, then, some dimensions are inactive (these dimensions form the null space and the nullity of H) and for these dimensions the rate is zero hence they can be excluded from the realization of Figure 9. In the sequel, we will present simulations where we study the reverse-waterfilling in the spatial domain under a certain distortion constraint studied in this paper.*

**Pre/Post Filtered ECDQ with multiplicative factors for augmented multivariate Gauss–Markov sources and spatial reverse-waterfilling.** First, we consider a rank(H)—dimensional lattice quantizer $Q_{\mathrm{rank}(H)}\left(\cdot \right)$ [48] such that

$$\begin{array}{c} E\left\{z_{t}z_{t}^{T}\right\}=\Sigma_{v^{c}},\;\Sigma_{v^{c}}≻0, \end{array}$$

where $z_{t}\in R^{\mathrm{rank}\left(H\right)}$ is a random dither vector generated both at the encoder and the decoder independent of the input signals ${\hat{x}}_{t}$ and the previous realizations of the dither, uniformly distributed over the basic Voronoi cell of the rank(H)—dimensional lattice quantizer $Q_{\mathrm{rank}(H)}\left(\cdot \right)$ such that $v_{t}^{c}∼Unif\left(0;\Sigma_{v_{t}^{c}}\right)$. At the encoder the lattice quantizer quantize $H{\hat{x}}_{t}+z_{t}$, that is, $Q_{\mathrm{rank}(H)}\left(H{\hat{x}}_{t}+z_{t}\right)$, where ${\hat{x}}_{t}$ is given by (66). Then, the encoder applies conditional entropy coding to the output of the quantizer and transmits the output of the entropy coder. At the decoder the coded bits are received and the output of the quantizer is reconstructed, i.e., $Q_{\mathrm{rank}\left(H\right)}\left(H{\hat{x}}_{t}+z_{t}\right)$. Then, it generates an estimate by subtracting $z_{t}$ from the quantizer’s output and multiplies the result by $I_{\mathrm{rank}\left(H\right)}$ ($I_{\mathrm{rank}\left(H\right)}$ denotes the identity matrix with dimensions according to the rank of *H*. This identity matrix can be excluded but we include it here for completeness.) as follows:

(73) $$\begin{array}{c} y_{t}=I_{\mathrm{rank}\left(H\right)}\left(Q_{\mathrm{rank}\left(H\right)}\left(H{\hat{x}}_{t}+z_{t}\right)-z_{t}\right), \end{array}$$

Performance. The coding rate at each instant of time of the conditional entropy of the MMSE quantizer is given by

(74) $$\begin{array}{cc} H(Q_{\mathrm{rank}\left(H\right)}|z_{t}) & =I(H{\hat{x}}_{t};H{\hat{x}}_{t}+v_{t}^{c})\\ \; & \overset{\left(a\right)}{=}I\left(H{\hat{x}}_{t};H{\hat{x}}_{t}+v_{t}\right)+D(v_{t}^{c}\left|\right|v_{t})-D(H{\hat{x}}_{t}+v_{t}^{c}||H{\hat{x}}_{t}+v_{t})\\ \; & \overset{\left(b\right)}{\leq }I\left(H{\hat{x}}_{t};H{\hat{x}}_{t}+v_{t}\right)+D(v_{t}^{c}\left|\right|v_{t})\\ \; & \overset{\left(c\right)}{\leq }I\left(H{\hat{x}}_{t};H{\hat{x}}_{t}+v_{t}\right)+\frac{\mathrm{rank}\left(H\right)}{2}\log\left(2\pi eG_{\mathrm{rank}\left(H\right)}\right)\\ \; & \overset{\left(d\right)}{\leq }I\left(x_{t};y_{t}|y^{t-1}\right)+\frac{\mathrm{rank}\left(H\right)}{2}\log\left(2\pi eG_{\mathrm{rank}\left(H\right)}\right) \end{array}$$

where $v_{t}^{c}\in R^{\mathrm{rank}\left(H\right)}$ is the (uniform) coding noise in the ECDQ scheme and $v_{t}$ is the corresponding Gaussian counterpart; (a) follows because the two random vectors $v_{t}^{c},v_{t}$ have the same second moments hence we can use the identity $D(x||x^{\prime })=h\left(x^{\prime }\right)-h\left(x\right)$; (b) follows because $D(H{\hat{x}}_{t}+v_{t}^{c}||H{\hat{x}}_{t}+v_{t})\geq 0$; (c) follows because the divergence of the coding noise from Gaussianity is less than or equal to $\frac{\mathrm{rank}\left(H\right)}{2}\log\left(2\pi eG_{\mathrm{rank}\left(H\right)}\right)$[47] where $G_{\mathrm{rank}\left(H\right)}$ is the dimensionless normalized second moment of the lattice ([44], Definition 3.2.2); (d) follows from data processing properties, namely, $I\left(x_{t};y_{t}|y^{t-1}\right)\overset{\left(*\right)}{=}I\left(x_{t};y_{t}|y_{t-1}\right)\overset{\left(**\right)}{=}I\left({\hat{x}}_{t};{\hat{y}}_{t}\right)\overset{\left(***\right)}{\geq }I\left(H{\hat{x}}_{t};H{\hat{x}}_{t}+v_{t}\right)$ where (*) follows from the realization of (71), (**) follows from the fact that ${\hat{x}}_{t}$ and ${\hat{y}}_{t}$ (obtained by (67)) are independent of $y_{t-1}$, and (***) is a consequence of data processing inequality since $\left(H{\hat{x}}_{t}+v_{t}\right)↔{\hat{x}}_{t}↔H{\hat{x}}_{t}$. Under the assumption that the clocks of the entropy encoder and entropy decoder in the ECDQ scheme are synchronized, then, the total coding rate is obtained as follows

(75) $$\begin{array}{cc} \sum_{t=1}^{n}R_{t}\leq \sum_{t=0}^{n}(H\left(Q_{\mathrm{rank}(H)}|z_{t}\right) & \overset{\left(e\right)}{\leq }\sum_{t=0}^{n}I\left(x_{t};y_{t}|y^{t-1}\right)+\frac{(n+1)\mathrm{rank}\left(H\right)}{2}\log\left(2\pi eG_{\mathrm{rank}\left(H\right)}\right)\\ \; & \overset{\left(f\right)}{=}\frac{1}{2}\sum_{t=0}^{n}\log_{2}\frac{|\Lambda_{t}|}{|\Delta_{t}|}+\frac{(n+1)\mathrm{rank}\left(H\right)}{2}\log\left(2\pi eG_{\mathrm{rank}(H)}\right), \end{array}$$

where (e) follows from (74); (f) follows from the derivation of Lemma 2.

The previous analysis yields the following theorem.

**Theorem** **4**  (Achievability bound on (11)–(13)). *Suppose that $\Delta_{t}=\Delta ,\;\forall t$ and assume that the users 1,2 source models (1) are driven by Gaussian noise processes. Then, the augmented state space source model in (3) ensures the following achievability bounds on (11)–(13), as follows*

(76) $$\begin{array}{cc} & R_{\mathrm{pd}}^{c}\left(D\right)\leq R_{\mathrm{pd}}^{LB}\left(D\right)+\frac{\mathrm{rank}(H)}{2}\log\left(2\pi eG_{\mathrm{rank}(H)}\right) \end{array}$$

(77) $$\begin{array}{cc} & R_{\mathrm{joint}}^{c}\left(D^{*}\right)\leq R_{\mathrm{joint}}^{LB}\left(D^{*}\right)+\frac{\mathrm{rank}(H)}{2}\log\left(2\pi eG_{\mathrm{rank}(H)}\right), \end{array}$$

(78) $$\begin{array}{cc} & R^{c}\left(D_{\mathrm{cov}}\right)\leq R^{LB}\left(D_{\mathrm{cov}}\right)+\frac{\mathrm{rank}(H)}{2}\log\left(2\pi eG_{\mathrm{rank}(H)}\right). \end{array}$$

**Proof.**  Under the conditions of the Theorem and the ECDQ scheme that leads to (75), the RHS terms in (76)–(78) are all constants. Then, taking the limit in both sides of (76)–(78) and then the appropriate infimization (minimization) constraint sets, the result follows. □

We wish to point out the following for Theorem 4.

**Remark** **11**  (Comments on Theorem 4).

**(1)**  The ECDQ that leads to (75) is not the same as the standard symmetric ECDQ scheme for scalar-valued processes, i.e., when the coefficient H breaks into two pre and post additive noise channel scalings that tune the MSE distortion (see, e.g., [44,47]). In our pre-post scaled ECDQ scheme we take asymmetric coefficients based on the realization of Figure 9. This leads to a coarser lattice than the one used for the unscaled ECDQ (for details see for instance [44]).
**(2)**  Since the upper bound essentially relies on the obtained lower bound for all (76)–(78), this means that similar observations can be made. For instance, if $D<D_{{}^{T}}$, then, (77) recovers (76), i.e., the asymptotically averaged total distortion constraint is inactive. Moreover, we cannot claim tightness of the achievability bound in (78) because the lower bound is already non-tight.

Next, we give an example where we compare the RL gap for various distortion levels of the achievability bound obtained in Theorem 4 and the lower bound obtained in Theorem 2 for the operational causal RDF with joint distortion constraints.

**Example** **4**  (RL gap of achievability and lower bounds). *In this example, we plot lower and upper bounds on the operational causal RDF subject to joint distortion constraints using the bounds derived in (50) and (77), respectively. We first consider the same input data assumed in Example 1 for users 1,2. Then, we proceed to compute the lower bound via (50) and the achievability bound in (77). After the first numerical study, we consider another one for which we only change the Gaussian noise process for both users 1,2, as follows*

(79) $$\begin{array}{c} \left(\Sigma_{w^{1}},\;\Sigma_{w^{2}}\right)=\left(\left[\begin{array}{cc} 1 & 0.5\\ 0.5 & 1 \end{array}\right],\;\left[\begin{array}{ccc} 1.4039 & 0.6034 & 0.5165\\ 0.6034 & 0.9563 & 0.7682\\ 0.5165 & 0.7682 & 0.6620 \end{array}\right]\right). \end{array}$$

**Figure 10:**
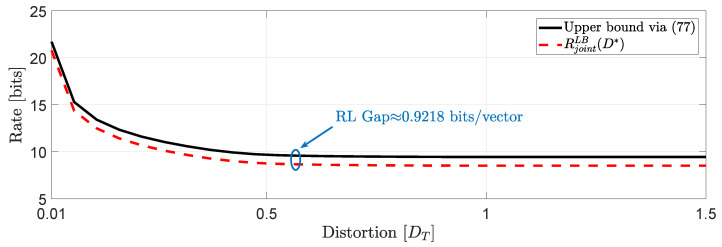
Comparison of lower and upper bounds on $R_{\mathrm{joint}}^{c}\left(D\right)$ when *H* is full rank.

*Using the data of Example 1, and the same $D_{{}^{T}}$ and $D_{ii},\;i=1,2,3,4,5$, we obtain the plots of Figure 10. For this study, we have used a Schläfli lattice (for details on this lattice see, e.g., [48]). ${\tilde{D}}_{5}$ with a dimensionless normalized second moment of the lattice $G_{5}\approx 0.0756$ bits. In this example H is always full rank and the RL gap is constant at 0.9218 bits/augmented vector.*

**Figure 11:**
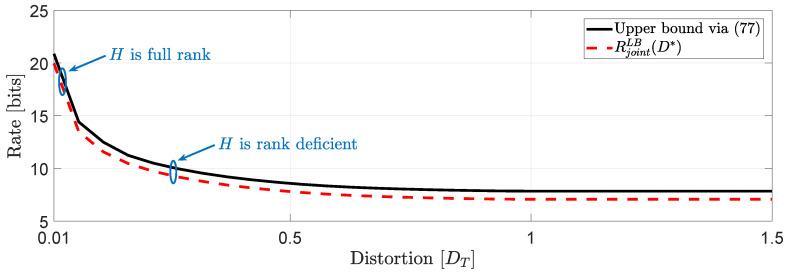
Comparison of the lower and upper bounds on $R_{\mathrm{joint}}^{c}\left(D\right)$ when *H* is rank deficient.

*For the second example, we obtain the plots of Figure 11. For this study, we have used the dimensionless normalized second moment of a Schläfli lattice ${\tilde{D}}_{5}$ for the full rank case and for the rank deficient cases a Schläfli lattice $D_{4}$ with a dimensionless normalized second moment of the lattice $G_{4}\approx 0.0766$ bits. Similar to the first study, when H is always full rank, the RL gap is 0.9218 bits/augmented vector whereas for H rank deficient the RL gap is 0.7754 bits/augmented vector.*

### 3.3. Bounding (11)–(13) via a DPCM-based ECDQ for Non-Gaussian Noise Processes

Similar to Lemma 4 where linear policies are the benchmark to derive lower bounds on (11)–(13), in this subsection we derive upper bounds on (11)–(13) using the linear test channel realization in Figure 9 and the DPCM-based ECDQ scheme of Section 3.1 and Section 3.2.

Next, we state the following theorem.

**Theorem** **5**  (Achievability bound on (11)–(13) for additive non-Gaussian noise process). *Suppose that $\Delta_{t}=\Delta ,\;\forall t$ and assume that the users 1,2 source models (1) are driven by non-Gaussian noise processes. Then, the augmented state space source model in (3) ensures the following achievability bounds on (11)–(13), as follows*

(80) $$\begin{array}{cc} & R_{\mathrm{pd}}^{c}\left(D\right)\leq R_{\mathrm{pd}}^{LB,linear}\left(D\right)+\frac{\mathrm{rank}(H)}{2}\log\left(2\pi eG_{\mathrm{rank}(H)}\right)+D(\hat{x}\left|\right|{\hat{x}}^{G}) \end{array}$$

(81) $$\begin{array}{cc} & R_{\mathrm{joint}}^{c}\left(D^{*}\right)\leq R_{\mathrm{joint}}^{LB,linear}\left(D^{*}\right)+\frac{\mathrm{rank}(H)}{2}\log\left(2\pi eG_{\mathrm{rank}(H)}\right)+D(\hat{x}\left|\right|{\hat{x}}^{G}), \end{array}$$

(82) $$\begin{array}{cc} & R^{c}\left(D_{\mathrm{cov}}\right)\leq R^{LB,linear}\left(D_{\mathrm{cov}}\right)+\frac{\mathrm{rank}(H)}{2}\log\left(2\pi eG_{\mathrm{rank}(H)}\right)+D(\hat{x}\left|\right|{\hat{x}}^{G}). \end{array}$$

*where $D(\hat{x}||{\hat{x}}^{G})$ is the KL divergence between the residual source ${\hat{x}}_{t}$ assuming linear policies and the Gaussian residual source ${\hat{x}}_{t}^{G}∼N\left(0;\Lambda \right)$ in Figure 9.*

**Proof.**  We only prove (80) because (81) and (82) follow similarly. In addition, in parts we sketch the derivation because it is clear from the previous results. From Lemmas 4 and 3, we can easily obtain the following lower bound (similar to SLB [32])

(83) $$\begin{array}{c} R_{\mathrm{pd}}^{c}\left(D\right)\geq R_{\mathrm{pd}}^{LB,linear}\left(D\right)-D(\hat{x}\left|\right|{\hat{x}}^{G}), \end{array}$$

where $D(\hat{x}||{\hat{x}}^{G})\geq 0$ is the discrepancy between the residual source ${\hat{x}}_{t}$ assuming linear policies and the optimal Gaussian residual source ${\hat{x}}_{t}^{G}∼N\left(0;\Lambda \right)$. From (83), we obtain

(84) $$\begin{array}{c} R_{\mathrm{pd}}^{LB,linear}\left(D\right)\leq R_{\mathrm{pd}}^{c}\left(D\right)+D(\hat{x}\left|\right|{\hat{x}}^{G}). \end{array}$$

Then, applying the DPCM-based ECDQ scheme based on the linear forward test channel realization of Figure 9 discussed in Section 3.1 and Section 3.2 we obtain (76). Since the coding scheme is obtained because we have assumed linear policies, $R_{\mathrm{pd}}^{LB}\left(D\right)$ will be replaced by $R_{\mathrm{pd}}^{LB,linear}\left(D\right)$. This completes the derivation. □

**Remark** **12**  (Comments on Theorem 5). *Clearly, Theorem 5 is a generalization of Theorem 4 under the assumption of the linear realization of Figure 9 with systems driven by additive i.i.d. non-Gaussian noise process. If in (80)–(82), we assume that the system is driven by additive i.i.d. Gaussian noise process, then, clearly $D(\hat{x}||{\hat{x}}^{G})=0$ and Theorem 5 recovers Theorem 4.*

## 4. Conclusions and Future Research

In this paper, we derived bounds on the OPTA of a two-user MIMO causal encoding and causal decoding problem (assuming the clocks of the encoder and the decoder to be synchronized). In our setup, each one of the users is described by a multivariate Markov source driven by additive i.i.d. noise process (possibly non-Gaussian) subject to three classes of spatio-temporal distortion constraints.

Although not directly pursued in this paper, all the results can be easily generalized to any finite number of users in Figure 1. Moreover, as a future research we aim to study the case of separate encoding for each user which will be a generalized version of a multi-user (distributed) source coding setup. Finally, due to the lack of insight of our results (mainly because we employed the general SDP solvers to compute our bounds) it makes sense to consider more specific setups and try to solve them using KKT conditions and, then, identify structural properties of matrices $\left(A,\;\Sigma_{w}\right)$ for which KKT conditions can be optimally solved.

## Notation

The following notation is used in this manuscript:

Symbol	Description
R	The set of real numbers
Z	The set of integers
$N_{0}$	The set of natural numbers including zero
$N_{0}^{n}$	The set {0,…,n} where $n\in N_{0}$
x∈R	Random variable
X	Alphabet for the random variable x
$x_{r}^{t}$	The sequence of random variables $\left(x_{r},x_{r+1},\ldots ,x_{t}\right)$, (r,t)∈Z×Z,r≤t
$x_{r}^{t}$	Sequence of the random variables realizations, where $x_{r}^{t}\in X_{r}^{t}$
$X_{r}^{t}$	$\times_{k=r}^{t}X_{k}$ with $X_{t}=X$
P(dx)	The probability of the RV x on X
P(dy|x)	The conditional distribution of RV y given x=x
⊗	compound product
⊕	Direct sum
$x\in R^{p\times 1}$	Column vector
$x^{T}\in R^{1\times p}$	Row vector
$K\in R^{p\times p}$	Square real matrix
$K^{T}\in R^{p\times p}$	Transpose of square real matrix
$K_{ii}$	Diagonal elements of matrix *K*
|K|	Determinant of *K*
rank(K)	Rank of *K*
trace(K)	Trace of *K*
$\mu_{K,i}$	the $i^{\mathrm{th}}$ eigenvalue of matrix *K*
$\Sigma_{x}$	The covariance of a random vector x
$\Sigma_{x}≻0$	Positive definite covariance matrix $\Sigma_{x}$
$\Sigma_{x}⪰0$	Positive semidefinite covariance matrix $\Sigma_{x}$
$\Sigma_{x}⪰\Sigma_{x^{\prime }}$	$\Sigma_{x}-\Sigma_{x^{\prime }}$ is positive semidefinite
$\Sigma_{x}≻\Sigma_{x^{\prime }}$	$\Sigma_{x}-\Sigma_{x^{\prime }}$ is positive definite
0	Null matrix
$I_{p}$	Identity matrix of dimension *p*
H(·)	Discrete entropy
h(·)	Differential entropy
$D(x||x^{\prime })$	KL Divergence of probability distribution P(x) with respect to probability distribution $P(x^{\prime })$
x∼N(0;Σ)	Gaussian random vector x with zero mean and covariance Σ
x∼Unif(0;Σ)	Uniformly distributed random vector x with zero mean and covariance Σ
$h^{G}\left(\cdot \right)$	Gaussian differential entropy
$R^{G}\left(\cdot \right)$	Gaussian information RDF
N(x)	The entropy power of random vector x
$\left|\right|\cdot {\left|\right|}_{2}$	Euclidean norm
E{·}	Expectation operator
${\left[\cdot \right]}^{+}$	max{0,·}
A↔B↔C	A,B,C form a Markov chain

## Abbreviations

The following abbreviations are used in this manuscript:

OPTA	Optimal Performance Theoretically Attainable
RDF	Rate distortion function
DPCM	Differential pulse coded modulation
ECDQ	Entropy coded dithered quantization
MSE	Mean-squared error
MMSE	Minimum MSE
RHS	Right Hand Side
LHS	Left Hand Side
i.i.d.	Independent Identically Distributed
a.s.	almost surely
KKT	Karush Kuhn Tucker
LMI	Linear matrix inequality
SDP	Semidefinite programming
KF	Kalman filter
EP	Entropy power
EPI	Entropy power inequalities
RL	Rate loss
SLB	Shannon Lower Bound

## Appendix A

**Proof of Lemma** **3.** We only prove **(1)** as both **(2)**, **(3)**, follow similarly. First, by assumptions of the theorem, $\Delta ≜\frac{1}{n+1}\sum_{t=0}^{n}\Delta_{t}$ for some finite *n* with Δ≻0 (sufficient condition for existence of a finite solution) and $B≜A^{T}\Sigma_{w}^{-1}A⪰0$. Moreover,

(A1) $$\begin{array}{cc} \frac{1}{n+1}\sum_{t=0}^{n}R_{t} & \overset{\left(a\right)}{\geq }\frac{1}{n+1}\sum_{t=0}^{n}I\left(x_{t};y_{t}|y^{t-1}\right)\\ & \overset{\left(b\right)}{=}\frac{1}{n+1}\frac{1}{2}\sum_{t=0}^{n}\log\frac{|\Lambda_{t}|}{|\Delta_{t}|}\\ & \overset{\left(c\right)}{=}\frac{1}{n+1}\left[\frac{1}{2}\log|\Lambda_{0}|-\frac{1}{2}\log\left|\Lambda_{n+1}\right|+\frac{1}{2}\sum_{t=0}^{n}\log\frac{|\Lambda_{t+1}|}{|\Delta_{t}|}\right]\\ & \overset{\left(d\right)}{=}\frac{1}{n+1}\left[\frac{1}{2}\log|\Lambda_{0}|-\frac{1}{2}\log\left|\Lambda_{n+1}\right|+\frac{1}{2}\sum_{t=0}^{n}\left[\log|\Sigma_{w}\left|+\log\right|\Delta_{t}^{-1}+B|\right]\right]\\ & \overset{\left(e\right)}{\geq }\frac{1}{n+1}\left[\frac{1}{2}\log\left|\Lambda_{0}\right|-\frac{1}{2}\log\left(\frac{{\left(\mathrm{trace}\left(A^{T}A\right)\mathrm{trace}\left(\hat{D}\right)+\mathrm{trace}\left(\Sigma_{w}\right))\right)}^{p}}{p}\right)\right]\\ & \;+\frac{1}{2}\frac{1}{n+1}\sum_{t=0}^{n}\left[\log|\Sigma_{w}\left|+\log\right|\Delta_{t}^{-1}+B|\right]\\ & \overset{\left(f\right)}{\geq }\frac{1}{n+1}\left[\frac{1}{2}\log\left|\Lambda_{0}\right|-\frac{1}{2}\log\left(\frac{{\left(\mathrm{trace}\left(A^{T}A\right)\mathrm{trace}\left(\hat{D}\right)+\mathrm{trace}\left(\Sigma_{w}\right))\right)}^{p}}{p}\right)\right]\\ & \;+\frac{1}{2}\log|\Sigma_{w}|+\frac{1}{2}\log\left|\Delta^{-1}+B\right|\\ & \overset{\left(g\right)}{\geq }\frac{1}{n+1}\left[\frac{1}{2}\log\left|\Lambda_{0}\right|-\frac{1}{2}\log\left(\frac{{\left(\mathrm{trace}\left(A^{T}A\right)\mathrm{trace}\left(\hat{D}\right)+\mathrm{trace}\left(\Sigma_{w}\right))\right)}^{p}}{p}\right)\right]+\frac{1}{2}\log\frac{\left|\Lambda \right|}{\left|\Delta \right|}, \end{array}$$

where (a) follows from Theorem 1 (see also Remark 2); (b) follows from Lemma 1, **(1)**, because $h^{G}\left(x_{t}|y^{t-1}\right)=\frac{1}{2}\log{\left(2\pi e\right)}^{p_{1}+p_{2}}\left|\Lambda_{t}\right|$ and $h^{G}\left(x_{t}|y^{t}\right)=\frac{1}{2}\log{\left(2\pi e\right)}^{p_{1}+p_{2}}\left|\Delta_{t}\right|$; (c) follows by reformulating the additive objective; (d) follows because $|\Lambda_{t+1}\left|\right|\Delta_{t}^{-1}|=|A\Delta_{t}A^{T}+\Sigma_{w}\left|\right|\Delta_{t}^{-1}\left|=\right|\Sigma_{w}\left(\Sigma_{w}^{-1}A\Delta_{t}A^{T}+I_{p_{1}+p_{2}}\right)\left|\right|\Delta_{t}^{-1}|\overset{\left(d1\right)}{=}|\Sigma_{w}\left(A^{T}\Sigma_{w}^{-1}A\Delta_{t}+I_{p_{1}+p_{2}}\right)\left|\right|\Delta_{t}^{-1}|\overset{\left(d2\right)}{=}|\Sigma_{w}||B+\Delta_{t}^{-1}|$ where (d1) follows from Weinstein–Aronszajn identity ([49], Corollary 18.1.2) and (d2) from standard determinant properties of square matrices of the same size; (e) follows from the inequalities

$$\begin{array}{cc} |\Lambda_{n+1}| & \overset{\left(e1\right)}{\leq }\frac{{\left(\mathrm{trace}\left(A\Delta_{n}A^{T}+\Sigma_{w}\right)\right)}^{p}}{p}\\ & \overset{\left(e2\right)}{\leq }\frac{{\left(\mathrm{trace}\left(A\Delta_{n}A^{T}\right)+\mathrm{trace}\left(\Sigma_{w}\right))\right)}^{p}}{p}\\ & \overset{\left(e3\right)}{\leq }\frac{{\left(\mathrm{trace}\left(A^{T}A\right)\mathrm{trace}\left(\Delta_{n}\right)+\mathrm{trace}\left(\Sigma_{w}\right))\right)}^{p}}{p}\\ & \overset{\left(e4\right)}{\leq }\frac{{\left(\mathrm{trace}\left(A^{T}A\right)\mathrm{trace}\left(\hat{D}\right)+\mathrm{trace}\left(\Sigma_{w}\right))\right)}^{p}}{p}, \end{array}$$

where (e1) follows because ${\left|K\right|}^{\frac{1}{p}}\leq \frac{\mathrm{trace}(K)}{p}$ for K≻0, (e2) follows from ([18], Ex. 12.14), (e3) follows from the cycling property of trace and ([18], Ex. 12.14), (e4) follows because $\mathrm{trace}\left(\Delta_{n}\right)\leq \mathrm{trace}\left(\hat{D}\right)$ (by definition); (f) follows because the term $\log|\Delta_{t}^{-1}+B|$ is convex with respect to $\Delta_{t}$ for B⪰0 (see, e.g., [50]) hence we can apply Jensen’s inequality ([19], Theorem 2.6.2); (g) follows because $\log|\Sigma_{w}|+\frac{1}{2}\log\left|\Delta^{-1}+B\right|$ can be rearranged to $\frac{\left|\Lambda \right|}{\left|\Delta \right|}$ where $\Lambda =A\Delta A^{T}+\Sigma_{w}$. Taking the limit in both sides in (A1), we observe that the first RHS term vanishes asymptotically. Finally, using the appropriate infimization constraints in both sides of the limiting objective functions in (A1), and because we assume sufficient conditions for existence of solution of the RHS term infimum is in fact minimum and the result follows. □

## Appendix B

**Proof of Theorem** **2.** Similar to the proof of Lemma 3, we only prove one case, i.e., **(1)**, as the following cases can be shown using the exact same way. By invoking matrix determinant lemma ([49], Theorem 18.1.1) in $R_{\mathrm{pd}}^{LB}\left(D\right)$, we obtain

(A2) $$\begin{array}{c} R_{\mathrm{pd}}^{\mathrm{na}}\left(D\right)=\min_{\begin{array}{c} 0≺\Delta ⪯\Lambda \\ \Delta_{ii}\leq D_{ii},\;i=1,\ldots ,p \end{array}}\frac{1}{2}\log|\Sigma_{w}|-\frac{1}{2}\log{\left|\Delta^{-1}-A{}^{T}\Sigma_{w}^{-1}A\right|}^{-1}. \end{array}$$

Next, we introduce a decision variable $Q_{1}≜\Delta^{-1}-A{}^{T}\Sigma_{w}^{-1}A$. Using the monotonicity of the determinant we can rewrite (A2) as

(A3) $$\begin{array}{c} R_{\mathrm{pd}}^{\mathrm{na}}\left(D\right)=\min_{\begin{array}{c} 0≺\Delta ⪯\Lambda \\ \Delta_{ii}\leq D_{ii},\;i=1,\ldots ,p\\ 0≺Q_{1}⪯{\left(\Delta^{-1}-A^{T}\Sigma_{w}^{-1}A\right)}^{-1} \end{array}}\frac{1}{2}\log|\Sigma_{w}|-\frac{1}{2}\log\left|Q_{1}\right|. \end{array}$$

Applying Woodbury matrix identity ([49], Theorem 18.2.8) in the inequality constraint $0≺Q_{1}⪯{\left(\Delta^{-1}-A{}^{T}\Sigma_{w}^{-1}A\right)}^{-1}$, we obtain

(A4) $$\begin{array}{c} 0≺Q_{1}⪯\Delta -\Delta A{}^{T}{\left(\Sigma_{w}+A\Delta A{}^{T}\right)}^{-1}A\Delta . \end{array}$$

From Theorem 2 we have $\Lambda =A\Delta A{}^{T}+\Sigma_{w}$, hence (A4) is equivalent to the LMI condition of (49). The decision variable is convex and there exists an optimal solution because Δ≻0. This completes the proof. □

## Appendix C

**Proof of Theorem** **3.** We only prove in detail **(1)** and sketch the proof for **(2)**, **(3)** because several steps are identical to **(1)**. First note that from Lemma 3 we have the bound in (43). Next, we simply show that the objective function can be lower bounded by a constant value (independent of the constraint set).

(A5) $$\begin{array}{cc} \frac{1}{2}\log\frac{\left|\Lambda \right|}{\left|\Delta \right|} & =\frac{1}{2}\log\left|A\Delta A^{T}+\Sigma_{w}\right|-\frac{1}{2}\log\left|\Delta \right|\\ & =\frac{\left(p_{1}+p_{2}\right)}{2}\log{\left|A\Delta A^{T}+\Sigma_{w}\right|}^{\frac{1}{p_{1}+p_{2}}}-\frac{\left(p_{1}+p_{2}\right)}{2}\log{\left|\Delta \right|}^{\frac{1}{p_{1}+p_{2}}}\\ & \overset{\left(a\right)}{\geq }\frac{\left(p_{1}+p_{2}\right)}{2}\log\left(|A\Delta A^{T}{|}^{\frac{1}{p_{1}+p_{2}}}+{\left|\Sigma_{w}\right|}^{\frac{1}{p_{1}+p_{2}}}\right)-\frac{\left(p_{1}+p_{2}\right)}{2}\log{\left|\Delta \right|}^{\frac{1}{p_{1}+p_{2}}}\\ & \overset{\left(b\right)}{=}\frac{\left(p_{1}+p_{2}\right)}{2}\log\left(|A^{T}{A|}^{\frac{1}{p_{1}+p_{2}}}{\left|\Delta \right|}^{\frac{1}{p_{1}+p_{2}}}+{\left|\Sigma_{w}\right|}^{\frac{1}{p_{1}+p_{2}}}\right)-\frac{\left(p_{1}+p_{2}\right)}{2}\log{\left|\Delta \right|}^{\frac{1}{p_{1}+p_{2}}}\\ & =\frac{\left(p_{1}+p_{2}\right)}{2}\log\left(|A^{T}{A|}^{\frac{1}{p_{1}+p_{2}}}+|\Sigma_{w}{|}^{\frac{1}{p_{1}+p_{2}}}{\left|\Delta \right|}^{-\frac{1}{p_{1}+p_{2}}}\right)\\ & \overset{\left(c\right)}{\geq }\frac{\left(p_{1}+p_{2}\right)}{2}\log\left(|A^{T}{A|}^{\frac{1}{p_{1}+p_{2}}}+{\left|\Sigma_{w}\right|}^{\frac{1}{p_{1}+p_{2}}}\frac{\left(p_{1}+p_{2}\right)}{\mathrm{trace}(\Delta )}\right) \end{array}$$

(A6) $$\begin{array}{c} \overset{\left(d\right)}{\geq }\frac{\left(p_{1}+p_{2}\right)}{2}\log\left(|A^{T}{A|}^{\frac{1}{p_{1}+p_{2}}}+{\left|\Sigma_{w}\right|}^{\frac{1}{p_{1}+p_{2}}}\frac{\left(p_{1}+p_{2}\right)}{D}\right) \end{array}$$

(A7) $$\begin{array}{c} \overset{\left(e\right)}{\geq }\frac{\left(p_{1}+p_{2}\right)}{2}\log\left(|A^{T}{A|}^{\frac{1}{\left(p_{1}+p_{2}\right)}}+\frac{\left(p_{1}+p_{2}\right)N\left(w\right)}{D}\right), \end{array}$$

where (a) follows from Minkowski’s determinant inequality ([18], Exercise 12.13); (b) follows from standard properties of determinants for square matrices of the same size; (c) follows from the reverse application of EPI (see, e.g., ([43], Equation (7))); (d) follows because $\mathrm{trace}\left(\Delta \right)\leq \mathrm{trace}\left(\hat{D}\right)\equiv D$; (e) follows from ([43], Equation (7)). The constant value in (A7) is well defined if $D\in \left(0,\frac{\left(p_{1}+p_{2}\right)N\left(w\right)}{1-|A^{T}{A|}^{\frac{1}{p_{1}+p_{2}}}}\right]$, where $N\left(w\right)=\frac{1}{2\pi e}2^{\frac{2}{p_{1}+p_{2}}h\left(w\right)}$ and h(w)>−∞. In **(2)** we follow similar steps to **(1)** but in inequality (d) we have instead $\mathrm{trace}\left(\Delta \right)\leq \min\left\{D_{{}^{T}},\mathrm{trace}\left(\hat{D}\right)\right\}\equiv D^{*}$. In **(3)** we follow similar steps until (A5). Afterwards, we leverage the fact that $\left|\Delta |\leq \right|D_{\mathrm{cov}}|$ to obtain instead of (A6) the following:

$$\begin{array}{c} \frac{\left(p_{1}+p_{2}\right)}{2}\log\left(|A^{T}{A|}^{\frac{1}{p_{1}+p_{2}}}+\frac{|\Sigma_{w}{|}^{\frac{1}{p_{1}+p_{2}}}}{|D_{\mathrm{cov}}{|}^{\frac{1}{p_{1}+p_{2}}}}\right). \end{array}$$

This completes the derivation. □
